# Multi-omics profiling of sodium-overload (NECSO) programs identifies NEK8 as a central driver of colorectal cancer progression through single-cell and spatial transcriptomics

**DOI:** 10.3389/fimmu.2026.1765055

**Published:** 2026-02-10

**Authors:** Yanchao Ji, Bo Yu, Xuedong Yin, Tianqi Yu, Xu Wu, Zongrui Zhao, Zheng Wang, Xiangjie Gao, Jiajun Zhao, Zhihao Fang, Yaxuan Wang, Yingnan Yu

**Affiliations:** 1 Department of General Surgery, Fourth Affiliated Hospital of Harbin Medical University, Harbin, China; 2 Department of Urology, The First Affiliated Hospital of Harbin Medical University, Harbin, China

**Keywords:** colorectal cancer, NEK8, single-cell RNA-seq, sodium-overload cell death, spatial transcriptomic, TRPM4

## Abstract

**Background:**

Colorectal cancer (CRC) is a leading cause of cancer-related death, often marked by intratumoral heterogeneity, immune evasion, and therapeutic resistance. Recent advancements in single-cell and spatial transcriptomics have enabled a deeper understanding of the tumor microenvironment (TME), revealing key insights into metabolic reprogramming and immune suppression. This study focuses on the role of sodium-overload cell death (NECSO) and its interaction with immune modulation in CRC pathogenesis.

**Methods:**

We employed an integrated multi-omic approach combining single-cell RNA-sequencing (scRNA-seq) and spatial transcriptomics (ST) to identify key NECSO-related genes in CRC. Through differential expression analysis and LASSO-Cox regression modeling, we developed a NECSO-based prognostic model. We validated our findings using TCGA and GEO datasets, assessing immune infiltration and spatial gene localization to determine the relationship between NEK8 and immune modulation.

**Results:**

Our NECSO-derived five-gene signature (NEK8, DRD4, EPHB2, CYTH2, ACOT11) effectively stratified CRC patients into high- and low-risk groups. High-risk patients exhibited reduced immune cell infiltration, particularly CD8^+^ T cells, and showed poorer survival outcomes. Single-cell and spatial data confirmed the upregulation of NECSO activity in malignant epithelial cells and its association with immune suppression. NEK8, a central driver in the NECSO program, was significantly overexpressed in CRC and correlated with poor prognosis. Functional assays revealed that NEK8 knockdown inhibited CRC cell proliferation, migration, and invasion, reinforcing its potential as a therapeutic target.

**Conclusions:**

The NECSO-derived signature provides a novel prognostic tool that integrates immune evasion and metabolic reprogramming in CRC. NEK8 plays a pivotal role in shaping the immune landscape and could serve as a biomarker for immunotherapy response, offering a pathway for personalized treatment strategies in precision oncology.

## 1 Introduction

Colorectal cancer (CRC) is the third most commonly diagnosed malignancy worldwide and a leading cause of cancer mortality (1). Although contemporary treatments—chemotherapy, surgical resection, targeted therapy, and immunotherapy—have improved survival, outcomes remain suboptimal because of marked intratumoral heterogeneity, therapeutic resistance, and the high recurrence of metastatic disease (2, 3). Advances in genomics and tumor-microenvironment (TME) research have refined our understanding of CRC pathogenesis. In the immunotherapy era, the cellular composition of the TME and its intercellular networks are pivotal and increasingly constitute actionable therapeutic targets (4). Nevertheless, molecular diversity and tumor heterogeneity drive substantial interpatient variability in treatment response. Accordingly, discovery of robust biomarkers and development of precise, mechanism-based strategies are imperative to enhance therapeutic efficacy and improve prognosis in CRC (5, 6).

Dysregulated sodium homeostasis is a salient pathogenic feature of the colorectal cancer (CRC) tumor microenvironment (TME) (7, 8). Evidence indicates that elevated intracellular sodium—by perturbing cellular metabolism, membrane potential, and ionic balance—promotes tumor initiation and progression (9). Under therapeutic pressure, sodium overload can become a key determinant of cancer-cell survival and drug resistance (10–12). With the description of sodium-overload cell death (NECSO), investigators have defined a death program triggered by pathological Na^+^ influx through dysregulated membrane routes, a rapid rise in intracellular sodium, and ensuing cell lysis (9). This process may be integral to CRC pathogenesis, progression, and treatment response. The transient receptor potential melastatin-4 (TRPM4) channel, a principal mediator of sodium entry, has been implicated in both NECSO and CRC development (9, 13). TRPM4 activation elevates intracellular sodium, fostering proliferative, migratory, and invasive phenotypes, and may likewise contribute to immune evasion and therapeutic resistance within the tumor context (14, 15).

Although sodium overload and the TRPM4 channel have been implicated in CRC, their mechanisms within the tumor microenvironment (TME) remain only partly defined. Single-cell RNA sequencing (scRNA-seq) now provides unprecedented resolution to map cellular heterogeneity and dynamic states in the TME, clarifying how discrete cell populations drive tumor initiation, progression, and therapy response (16, 17). Integrated with spatial transcriptomics (ST) and multimodal omics, these platforms permit spatial and functional dissection of sodium-overload and NECSO-related programs in CRC, yielding fresh insights and actionable hypotheses for individualized therapeutic strategies (18, 19). Several studies have successfully applied multi-omics strategies combined with structural or morphological data to uncover disease subtypes and their development, further validating the power of these approaches in disease research (20–22).

In this study, we analyzed transcriptomes spanning normal colonic tissue to colorectal cancer (CRC), focusing on genes implicated in sodium-overload cell death (NECSO). Using TCGA and GEO cohorts, we applied differential-expression and correlation filtering with TRPM4 as the index gene, delineating a panel of NECSO-related candidates. We then integrated single-cell RNA-seq (scRNA-seq) and spatial transcriptomics (ST) datasets from GEO to examine their expression patterns and spatial localization within the tumor microenvironment, emphasizing links to immune contexture and specific cellular lineages. Building on these datasets, we developed a NECSO-based prognostic risk score and optimized it with LASSO regression, demonstrating predictive potential for clinical outcomes in CRC. We also assessed associations between model genes and responses to immunotherapy and chemotherapy, providing insights to inform individualized therapeutic strategies.

## 2 Methods

### 2.1 Study design

**Figure 1:**
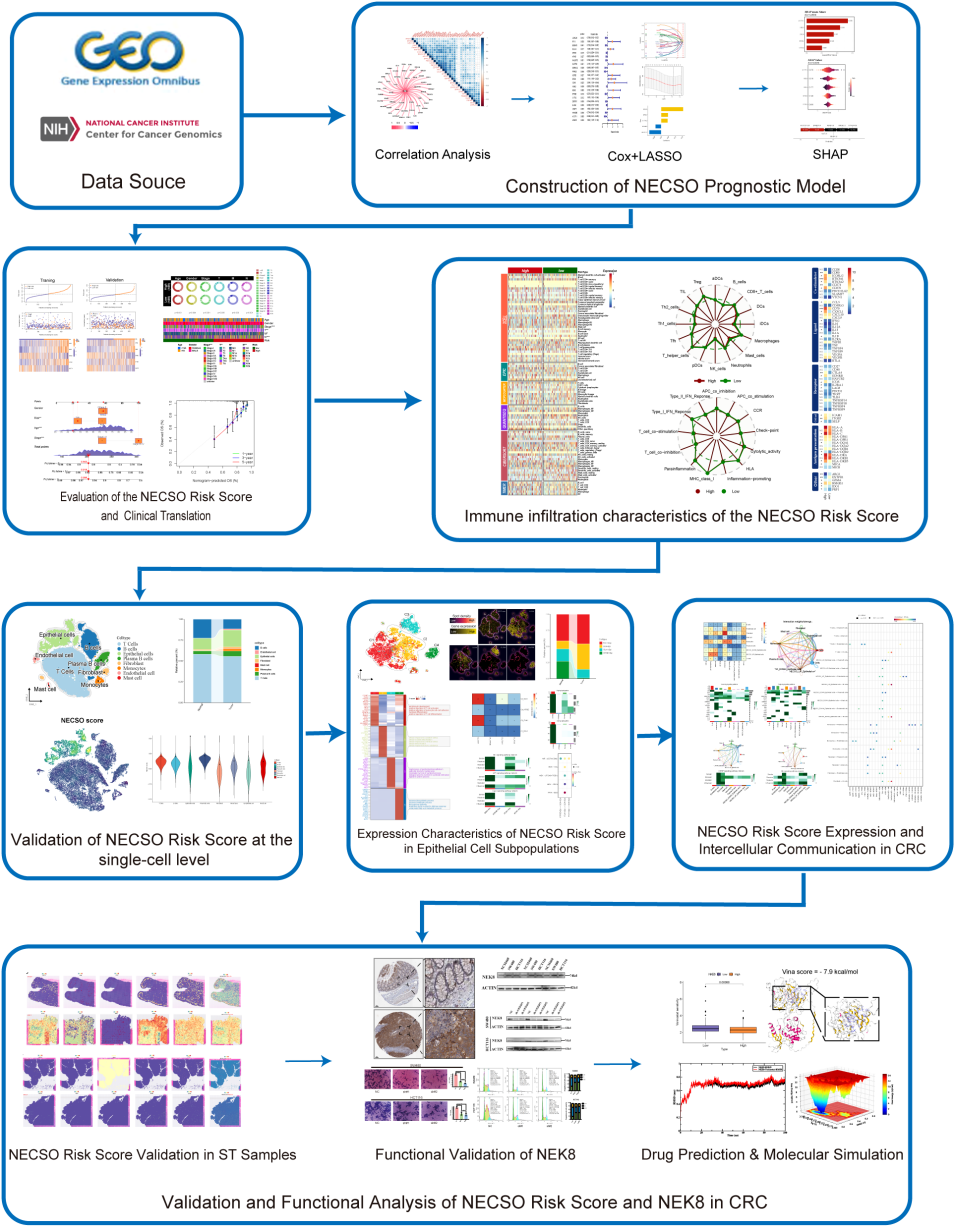
Flowchart of this study.

The study design is presented in **Figure 1**.

### 2.2 Data sources and preprocessing

We obtained RNA-seq data (TPM) and clinical annotations for TCGA-CRC from the Genomic Data Commons (https://portal.gdc.cancer.gov/) and converted all transcript abundances to TPM (transcripts per kilobase million) for downstream analyses, excluding genes with mean expression ≤ 0.1 TPM. The GEO microarray cohort GSE39582 served as an external validation set (23) (raw or normalized matrices available), with TCGA-CRC as the discovery cohort. We also curated CRC single-cell and spatial transcriptomic datasets from GEO: GSE200997 (16 tumors, 7 adjacent normals) (24)and GSE210038 (two tumors with paired adjacent normals) (25). Single-cell RNA-seq data were processed in Seurat v5.3.0 (26): expression matrices were imported with Read10X and assembled via CreateSeuratObject. Quality control retained cells with >200 and <3,000 detected genes, removed cells with mitochondrial content ≥20%, and excluded those with UMI counts (nCount_RNA) ≥20,000 (**Supplementary Figure 1A**). Batch effects were corrected with Harmony, followed by normalization and PCA (**Supplementary Figure 1B**). Using the top 20 PCs, we built a neighbor graph and clustered with FindNeighbors/FindClusters (resolution = 0.1, guided by clustree) (**Supplementary Figure 1C**), and visualized embeddings with RunUMAP. Cell types were annotated by canonical markers: B cells, endothelial cells, epithelial cells, fibroblasts, mast cells, monocytes, plasma B cells, and T cells. Differential expression was used FindMarkers with a two-sided Wilcoxon test (P < 0.05). NECSO scores were computed with AddModuleScore.For spatial transcriptomics, data were normalized with SCTransform, reduced and clustered with RunPCA/RunUMAP, and scored with AddModuleScore. Spatial distributions were visualized by SpatialFeaturePlot, defining spots with NECSO score > 0 as positive. Immunohistochemistry (IHC) images of colonic tissue were retrieved from the Human Protein Atlas (HPA).

### 2.3 Acquisition of NECSO-responsive genes

Using TRPM4 as the index gene (9), we identified NECSO-related candidates in colorectal cancer (CRC) transcriptomes with a two-stage “differential expression + correlation” workflow. TPM-scale matrices were standardized (duplicate gene symbols averaged), and low-abundance genes (row-mean ≤ 0.1 TPM) were removed. Samples were stratified into tumor and normal groups by TCGA barcodes. Stage 1 (differential expression): for each gene, two-sided Wilcoxon rank-sum tests (tumor vs normal) were applied; genes with P < 0.05 formed the evaluation set. Stage 2 (tumor-only correlation): Pearson correlations between TRPM4 and each evaluation-set gene were computed, requiring |r| > 0.30, P < 0.001, and tumor expression variability sd > 0.1. Genes satisfying both screens were designated TRPM4-associated (NECSO-related) candidates and carried forward for pathway enrichment, tumor–immune microenvironment analyses, and construction of the prognostic model. All analyses were performed in R.

### 2.4 Construction and validation of a prognostic model

In the training cohort, we first ran univariable Cox proportional-hazards models to identify genes associated with prognosis (P < 0.05). These candidates were then entered into a LASSO–Cox penalized regression (R package glmnet (27)) to achieve variable shrinkage and derive a parsimonious signature. The risk score was computed as


“Risk score”=∑_i (β_i×Exp_i ),

where ${\text{Exp}}_{i}$ is the expression level of gene i and $\beta_{i}$ its coefficient estimated by the LASSO model. Survival curves were generated with survminer (28), and time-dependent ROC analyses for 1-, 3-, and 5-year overall survival (OS) were performed using survROC (29) to assess discrimination. Model robustness was evaluated in the internal training set and an external independent cohort. To enhance interpretability, we conducted single-feature SHAP dependence analyses to quantify each gene’s marginal contribution to predicted risk, thereby strengthening the model’s potential clinical utility (30).

### 2.5 Functional and pathway enrichment analysis

We used the R package clusterProfiler (31) for functional annotation and pathway analysis of NECSO-related candidates, covering KEGG and the three Gene Ontology (GO) domains—biological process (BP), molecular function (MF), and cellular component (CC). Multiple testing was controlled with the Benjamini–Hochberg procedure; terms with FDR-adjusted P < 0.05 were deemed significant. We then performed gene set enrichment analysis (GSEA) (32, 33) to compare pathway activity between high- and low-risk groups using MSigDB-curated gene sets. Enrichments were ranked by FDR-adjusted P (< 0.05) and normalized enrichment score (NES). Transcriptome-wide, gene-set–level activity was further assessed with GSVA (34) using MSigDB gene sets (35) (http://www.gsea-msigdb.org/gsea/index.jsp); limma was applied in R to compare GSVA scores and delineate functional differences across samples.

### 2.6 Construction of a predictive nomogram

To visualize individualized prognostic predictions for CRC, we built a nomogram with the rms package incorporating sex, the model-derived risk score, age, and clinical stage. Each variable was assigned points proportional to its regression coefficient, and the total was mapped to predicted 1-, 3-, and 5-year overall survival (OS) probabilities. Clinical utility was assessed by decision curve analysis (DCA).

### 2.7 Immune infiltration analysis

To assess immune infiltration in colorectal cancer, we applied six deconvolution tools—xCell (36), EPIC, MCP-counter (37), QUANTISEQ, CIBERSORT (38), and TIMER (39)—to the TCGA-CRC RNA-seq cohort. These methods infer per-sample relative abundance or enrichment of immune-cell subsets from predefined marker signatures. Group differences stratified by the NECSO score were tested with the Wilcoxon rank-sum test, and multi-algorithm outputs were integrated in heatmaps for visualization. We also summarized seven classes of immunomodulators (40, 41), the canonical seven immune subtypes, and the immunophenotype score (IPS) (42) to refine immune profiling within the NECSO framework. Finally, IPS and tumor immune dysfunction and exclusion (TIDE) scores were used to estimate potential benefit from immunotherapy and gauge immune-evasion propensity; IPS and TIDE were retrieved from TCIA (https://tcia.at/home/ and the TIDE server (http://tide.dfci.harvard.edu/), respectively (43).

### 2.8 Tumor mutational burden analysis

We analyzed TCGA mutation annotation format (MAF) files with the R package maftools (44) and generated separate oncoplots (waterfall plots) for high- and low-risk groups. Using precomputed mutation frequencies, we displayed the top 15 recurrently mutated genes and overlaid clinical annotations indicating risk stratification.

### 2.9 Cell-cell communication analysis and cell fate trajectory analysis

To delineate crosstalk among epithelial subpopulations, we built cell–cell communication networks using CellChat (45), restricting analyses to “Secreted Signaling.” We estimated ligand–receptor probabilities with computeCommunProb, denoised interactions via filterCommunication, and aggregated signals to pathways using computeCommunProbPathway. Global network structure was summarized with aggregateNet, and pathway-specific patterns were visualized as heatmaps with netVisual_heatmap. To chart developmental trajectories, we applied Monocle2 (46): normalized matrices were imported, cluster DE genes (FDR-adjusted P < 0.05) served as ordering features, and default dimensionality reduction/cell ordering inferred pseudotime. Gene trends along pseudotime were z-score–standardized, smoothed, and visualized to highlight positively or negatively correlated sets. Lineage relationships during subtype bifurcation were inferred with Slingshot (47) using getLineages and getCurves to derive differentiation paths, which were projected onto UMAP for visualization. Cellular differentiation potential was quantified with CytoTRACE (48); the top 100 genes positively and negatively correlated with the CytoTRACE score were designated as immaturity and differentiation markers, respectively. CytoTRACE scores range from 0–1, where 0 denotes a more differentiated state and 1 a less differentiated state.

### 2.10 Molecular dynamics simulations in drug-target interaction analysis

We used the R package oncoPredict (v0.2) (49)—leveraging drug-response profiles and transcriptomes from the Broad Institute’s CTRP and the Sanger GDSC—to predict each patient’s responsiveness to multiple clinically relevant agents. To prioritize therapeutics, we performed molecular dynamics (MD) simulations to estimate binding free energies and characterize interactions between candidate small molecules and their protein targets, thereby assessing targetability. Two-dimensional ligand structures were retrieved from PubChem (https://pubchem.ncbi.nlm.nih.gov/), and three-dimensional receptor structures from the RCSB Protein Data Bank (PDB) (https://www.rcsb.org/). Initial protein–ligand docking used the CB-Dock2 web platform, and resulting complexes were subjected to MD evaluation of stability and binding interactions.

### 2.11 Molecular dynamics simulation

Molecular dynamics (MD) simulations were performed with GROMACS 2025.2. Small-molecule ligands were prepared in AmberTools 22 with the GAFF force field; geometries were protonated and RESP charges computed in Gaussian 16W. Parameters were written into the topology. Simulations ran at 300 K and 1 bar using the amber99sb-ildn protein force field and TIP3P water. Na^+^ counterions neutralized the system. After steepest-descent minimization, systems underwent NVT and NPT equilibration for 100,000 steps each (τ = 0.1 ps; ~100 ps total). Production trajectories were generated for 50,000,000 steps with a 2 fs timestep (100 ns total). Post-processing with GROMACS tools yielded root-mean-square deviation (RMSD), root-mean-square fluctuation (RMSF), solvent-accessible surface area (SASA), and radius of gyration (R_g). Binding thermodynamics were estimated by MM/GBSA, and conformational landscapes were profiled via free-energy surface analyses.

### 2.12 Cell culture and transfection

Cell lines NCM460, HCT-116, and SW480 were obtained from Haixing Biosciences (Suzhou, China). Cells were cultured in DMEM supplemented with 10% fetal bovine serum (FBS; Life Technologies, USA) and 100 U/mL penicillin–streptomycin at 37 °C in a humidified 5% CO_2_ incubator. A lentiviral RNAi vector targeting NEK8 (and a negative control) was provided by GeneChem (Shanghai, China). For infection, HCT-116 and SW480 cells were seeded in 6-well plates at ~2 × 10^5^ cells/well and allowed to adhere overnight. When cultures reached ~30–50% confluence the next day, cells were exposed to lentivirus encoding shNEK8 or the negative control (NC). Stable polyclonal lines were generated by selection in puromycin (2 mg/mL) for 2 weeks. Knockdown efficiency was evaluated 72 h postinfection by Western blotting. shRNA target sequences were: shNEK8#1, 5′-CGGGTGATTGCTACACTTT-3′; shNEK8#2, 5′-TGGTGATCATCAAGCAGAT-3′.

### 2.13 Western blot

Cells were lysed in RIPA buffer (Solarbio) with ultrasonication. Lysates were clarified by centrifugation at 14,000 × g for 15 min at 4 °C, and supernatants were collected. Protein concentration was measured by BCA. Equal protein amounts were resolved on 12.5% SDS–PAGE and transferred to PVDF membranes. Membranes were blocked in TBST with 5% skim milk and incubated with primary antibodies against NEK8 (Abcam, AB169406) and β-actin (ABclonal, AC026; 1:50,000), the latter as the loading control. After incubation with HRP-conjugated secondary antibody (ABclonal, AS014), signals were detected by enhanced chemiluminescence (ECL).

### 2.14 Quantitative real-time PCR

Total RNA was extracted from cultured cells with TRIzol (Invitrogen) per the manufacturer’s instructions. RNA concentration and purity were assessed by UV spectrophotometry; samples with an A260/A280 of 1.8–2.0 were accepted. Two micrograms of RNA were reverse-transcribed to cDNA using a commercial kit (TOYOBO, Osaka, Japan). Quantitative real-time PCR (qPCR) was performed with SYBR Green chemistry (Sevenbio, China). Primer sequences were: NEK8, forward 5′-CTTCGTGCAGATCCTGCTTG-3′ and reverse 5′-GGAGATGCCGAAATCACCGAT-3′; β-actin, forward 5′-CCATCGTCCACCGCAAAT-3′ and reverse 5′-GCTGTCACCTTCACCGTTCC-3′. Each 20 μL reaction contained 10 μL SYBR Green master mix, 1 μL forward primer (10 μM), 1 μL reverse primer (10 μM), 2 μL cDNA, and 6 μL nuclease-free water. β-actin served as the internal control. All samples were run in technical triplicate, and each experiment was independently repeated at least three times. Relative expression was calculated using the $2^{-\text{ΔΔ}C_{t}}$ method.

### 2.15 CCK-8 and colony-formation assays

To assess proliferation, CRC cells transduced with sh-NC or sh-NEK8 were seeded in 96-well plates. Growth was quantified with the Cell Counting Kit-8 (CCK-8; Solarbio, China) per the manufacturer’s protocol, and optical density (OD) was read at 450 nm. For colony formation, transduced cells were plated in 6-well plates at 1,000 cells/well and cultured for 10 days in RPMI-1640 with 10% fetal bovine serum. Colonies were fixed in methanol, stained with 1% crystal violet (Beyotime, China), imaged, and analyzed.

### 2.16 Wound-healing and transwell assays

For the wound-healing assay, a linear scratch was made across confluent monolayers with a pipette tip, and cells were cultured in serum-free medium. Wound closure was imaged and quantified at 48 h. Cell migration was assessed in 24-well Transwell chambers with 8-µm polycarbonate membranes. Matrigel (100 μL; 1:8 in serum-free medium) was spread on the upper chamber and allowed to solidify. Cell suspensions were added to the upper chamber, and the lower chamber contained medium with 10% fetal bovine serum. At assay end, membranes were rinsed twice in PBS, fixed in methanol, and stained with 0.5% crystal violet. Cells remaining on the upper surface were gently removed with a cotton swab, and cells that migrated to the underside were counted in predefined fields. All incubations were at 37 °C in a humidified 5% CO_2_ atmosphere.

### 2.17 Flow cytometry assay

HCT-116 and SW480 cells were fixed in ice-cold 70% ethanol for 12 h, then incubated with RNase at 37 °C for 30 min. Nuclei were stained with a propidium iodide (PI) kit (Sevenbio, China); PI staining was performed at 4 °C per the manufacturer’s instructions. Cell-cycle distribution was analyzed by flow cytometry within 1 h of staining. Data were processed and modeled with ModFit LT (Verity Software, Topsham, ME) and FlowJo v10 (Treestar, Ashland, OR).

### 2.18 Statistical analysis

For normally distributed variables, between-group differences were tested with the unpaired Student’s t-test; for non-normal data, the Wilcoxon rank-sum test was used. For multi-group comparisons, one-way ANOVA was applied to parametric data and the Kruskal–Wallis test to nonparametric data. Two-sided Fisher’s exact tests were used for contingency tables. All analyses were conducted in R (v4.5.0), GraphPad Prism (v9.5.0), and Python (v3.8).

## 3 Results

### 3.1 NECSO-related genes and construction of prognostic model

**Figure 2:**
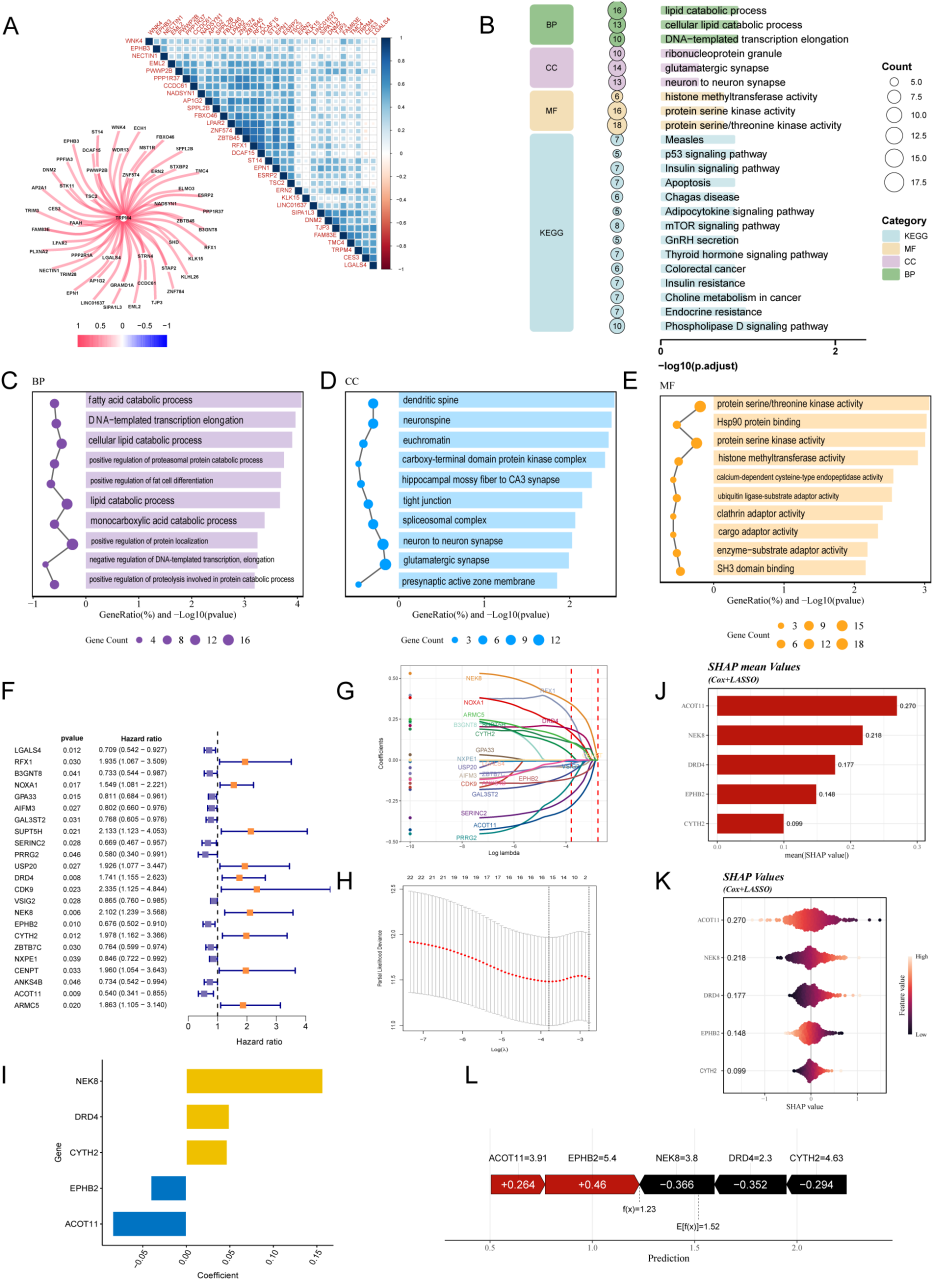
NECSO gene discovery, functional enrichment, and construction/interpretation of the prognostic model. **(A)** TRPM4 co-expression analysis in TCGA-CRC: top, pairwise correlation heatmap for the top 50 TRPM4-associated genes; bottom, gene–gene correlation network (color scale denotes Pearson’s *r*; blue, negative; red, positive). **(B)** Overview bubble plot of significant pathways across GO domains—biological process (BP), cellular component (CC), molecular function (MF)—and KEGG (bubble size = gene count; x-axis = –log10 adjusted *P*). **(C–E)** Dot plots of GO enrichment results: **(C)** BP, **(D)** CC, **(E)** MF (ranked by adjusted *P* and gene ratio). **(F)** Univariable Cox regression forest plot showing hazard ratios (HRs), 95% CIs, and *P* values for candidate genes. **(G)** LASSO–Cox coefficient paths as a function of log(λ). **(H)** Tenfold cross-validation curve identifying the optimal λ by the minimum-error and 1-SE criteria. **(I)** Bar plot of LASSO-selected coefficients for the five-gene signature (ACOT11, EPHB2, CYTH2, DRD4, NEK8). **(J)** Mean SHAP values quantifying each feature’s average contribution to risk prediction. **(K)** SHAP beeswarm plot showing the distribution of SHAP values per gene (color encodes expression from low to high) and their directional impact on predictions. **(L)** SHAP waterfall plot illustrating gene-wise contributions to the final prediction for a representative sample.

We leveraged TCGA-CRC expression data to examine the putative role of TRPM4 in sodium-overload cell death (NECSO). TRPM4—a Na^+^-conducting member of the transient receptor potential melastatin subfamily—is broadly expressed in heart, brain, and immune cells, where it modulates membrane potential and ionic homeostasis. Aberrant TRPM4 activation has been linked to hypoxia, energy depletion, and sodium-overload–related cell death (9). Using Pearson correlation, we quantified associations between TRPM4 and all other genes, identifying 359 transcripts significantly correlated with TRPM4 (**Supplementary Table 1**), which we designated as NECSO-related genes. We then generated a heatmap and gene–gene interaction network for the top 50 correlates (**Figure 2A**). TRPM4 showed robust positive correlations with PP1R37, NADSY1, and AP1G2, suggesting co-involvement in TRPM4-mediated NECSO and a role in CRC initiation and progression, whereas a subset of genes was negatively correlated, consistent with inhibitory regulation of TRPM4-linked functions. KEGG analysis (**Supplementary Table 2**) revealed significant enrichment across pathways governing lipid metabolism, the p53 axis, and insulin signaling, indicating that sodium overload may promote cell death by perturbing lipid programs, modulating cell-cycle control, and altering metabolic balance (**Figure 2B**). Gene Ontology enrichment (**Supplementary Table 3**) likewise showed overrepresentation across BP, CC, and MF domains: fatty-acid and cellular lipid catabolic processes (BP) linked to lipid metabolism; dendritic spines and neuronal synapses (CC) indicated synaptic interfaces; and protein serine/threonine kinase activity and Hsp90 binding (MF) implicated signaling and stress responses (**Figures 2C–E**). To prioritize clinically relevant NECSO genes, we performed univariable Cox analysis followed by LASSO regression (**Figures 2F–H**), yielding a five-gene signature. A NECSO risk index derived from these genes (**Figure 2I**; **Supplementary Table 4**) was interpreted with SHAP modeling. Mean SHAP values (**Figure 2J**) indicated substantial contributions from ACOT11, NEK8, DRD4, CYTH2, and EPHB2; distribution plots (**Figure 2K**) showed that expression variability in these genes materially influenced the index. The SHAP waterfall plot (**Figure 2L**) showed positive contributions from ACOT11 (+0.264) and EPHB2 (+0.460) and negative contributions from NEK8 (−0.366), DRD4 (−0.352), and CYTH2 (−0.294). The final prediction was 1.23 relative to an expected baseline E[f(x)]=1.52, suggesting heterogeneous roles of these genes within the NECSO program that may inform prognosis.

### 3.2 Clinical application and predictive performance evaluation of the NECSO risk score

**Figure 3:**
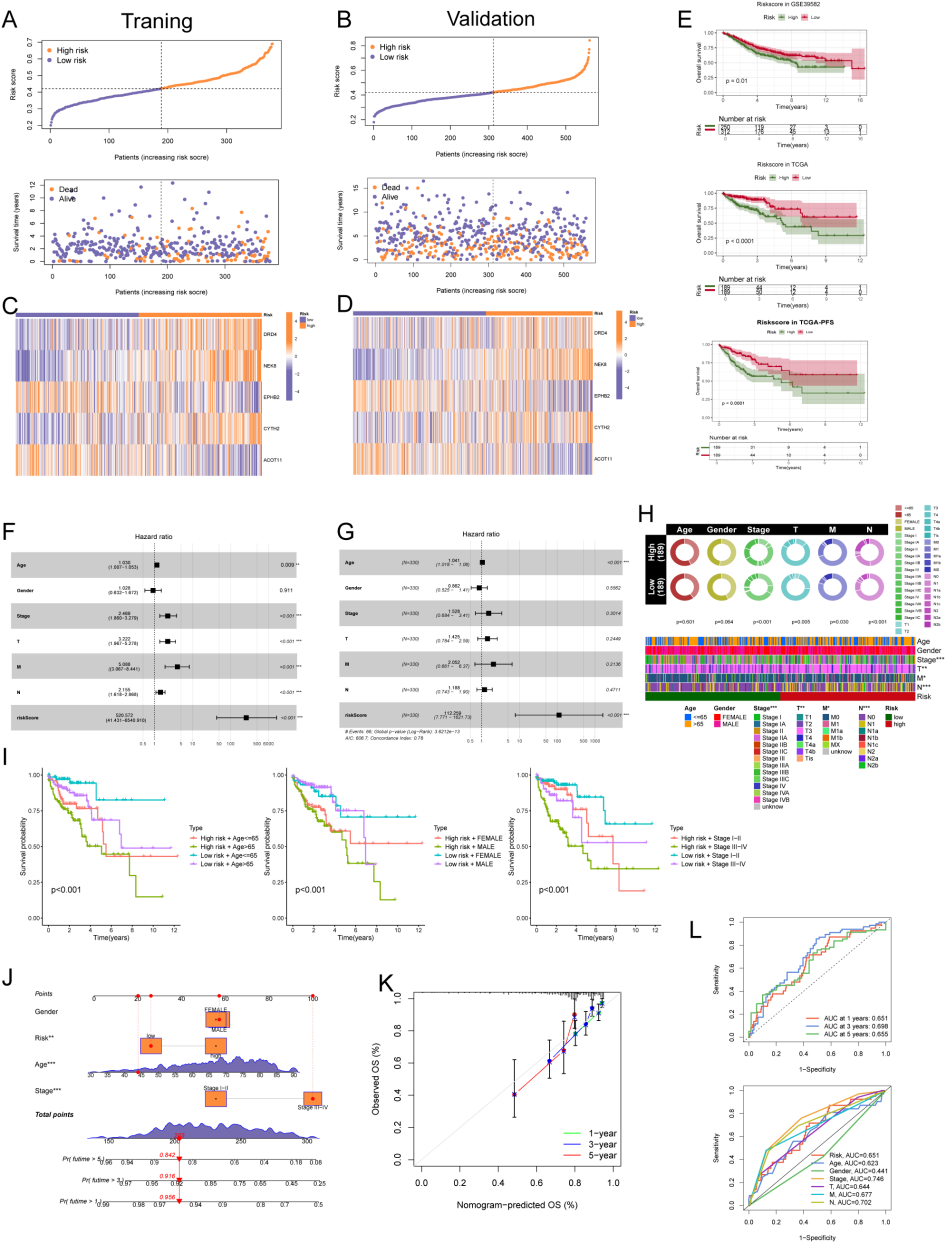
Performance and clinical utility of the NECSO-based five-gene prognostic signature. **(A)** Training cohort: ranked risk scores (top) and survival status (bottom) for each patient dichotomized at the median (orange, high risk; blue, low risk). **(B)** Validation cohort (GSE39582): ranked risk scores (top) and survival status (bottom) using the same procedure. **(C, D)** Heatmaps of signature-gene expression across risk strata in the training **(C)** and validation **(D)** cohorts (rows: DRD4, NEK8, EPHB2, CYTH2, ACOT11). **(E)** Kaplan–Meier curves comparing overall survival (GSE39582, TCGA) and progression-free survival (TCGA-PFS) between high- and low-risk groups; shaded areas denote 95% CIs. **(F)** Univariable Cox regression: forest plot of hazard ratios (HRs), 95% CIs, and *P* values for age, sex, stage, T/M/N categories, and the risk score. **(G)** Multivariable Cox regression, including the above covariates, the risk score remains an independent prognostic factor. **(H)** Clinical association summary: circular bar plots and an integrated heatmap showing distributions of age, sex, overall stage, and T/M/N categories across risk strata. **(I)** Stratified Kaplan–Meier analyses demonstrating inferior survival for high-risk patients within age-defined, sex-defined, and stage-defined subgroups. **(J)** Nomogram integrating sex, risk score, age, and stage to estimate individualized probabilities of 1-, 3-, and 5-year overall survival. **(K)** Calibration curves showing agreement between nomogram-predicted and observed survival at 1, 3, and 5 years. **(L)** Top: time-dependent ROC curves for 1-, 3-, and 5-year overall survival based on the risk score. Bottom: ROC comparison of the risk score versus clinical variables (age, stage, T/M/N, sex).

Using coefficients for five NECSO-related genes (DRD4, NEK8, EPHB2, CYTH2, ACOT11), we built a prognostic risk-score model and dichotomized patients at the median into high- and low-risk groups. Individual risk was calculated as *“* Risk score"=∑_i (β_i×Exp_i ), where ${\text{Exp}}_{i}$ is the expression of gene i and $\beta_{i}$ its LASSO-derived coefficient. TCGA-CRC served as the training set and GSE39582 as the validation set. High-risk patients had significantly shorter survival than low-risk patients (**Figure 3A**). Concordant patterns of risk-score distributions and survival status in both cohorts supported model stability (**Figure 3B**). Heatmaps showed marked expression differences across strata for DRD4, NEK8, and EPHB2, among others (**Figures 3C, D**). We further evaluated performance by Kaplan–Meier analyses in GSE39582, TCGA, and TCGA-PFS: high risk consistently predicted worse outcomes (GSE39582, p = 0.01; TCGA, p < 0.0001) and shorter progression-free survival (TCGA-PFS, p < 0.0001) (**Figure 3E**). In univariable Cox models, age (HR = 1.030, p = 0.009), overall stage (HR = 2.469, p < 0.001), T stage (HR = 3.222, p < 0.001), M stage (HR = 5.088, p < 0.001), N stage (HR = 2.155, p < 0.001), and the risk score (HR = 520.572, p < 0.001) were significant, whereas sex was not (HR = 1.028, p = 0.911) (**Figure 3F**). In multivariable models, the risk score (HR = 112.259, p < 0.001) remained the sole independent prognostic factor; age also retained significance (HR = 1.041, p < 0.001), while stage and T/M/N categories were not significant (**Figure 3G**). Clinical characteristics differed by risk group: age and sex were comparable (p = 0.601 and 0.064), whereas overall stage, T stage, M stage, and N stage were skewed toward advanced disease in the high-risk group (Stage III/IV, T3/T4, M1, N1/N2) and earlier disease in the low-risk group (Stage I/II, T1/T2, M0, N0) (p < 0.001, 0.005, 0.030, < 0.001). A composite heatmap underscored the link between clinical staging and risk (**Figure 3H**). Stratified Kaplan–Meier curves showed poorer survival for high-risk patients irrespective of age (>65 vs ≤65 years, both p < 0.001), sex (male or female, both p < 0.001), or stage (early vs advanced, both p < 0.001) (**Figure 3I**). A nomogram integrating sex, risk score, age, and stage (**Figure 3J**) yielded individualized prognostic scores and survival probabilities; high-risk patients accrued higher points, with predicted probabilities of surviving beyond 5, 3, and 1 years of 0.842, 0.916, and 0.956. Calibration plots showed good agreement between predicted and observed survival at 1, 3, and 5 years (**Figure 3K**). Time-dependent ROC analyses produced AUCs of 0.651 (1 year), 0.698 (3 years), and 0.655 (5 years). Compared with other variables, the risk score achieved an AUC of 0.651, while age (AUC = 0.823) and overall stage (AUC = 0.746) showed higher discrimination; sex (AUC = 0.441), T stage (AUC = 0.644), M stage (AUC = 0.677), and N stage (AUC = 0.702) contributed less but provided complementary information (**Figure 3L**).

### 3.3 Analysis of immune microenvironment characteristics based on NECSO risk score

**Figure 4:**
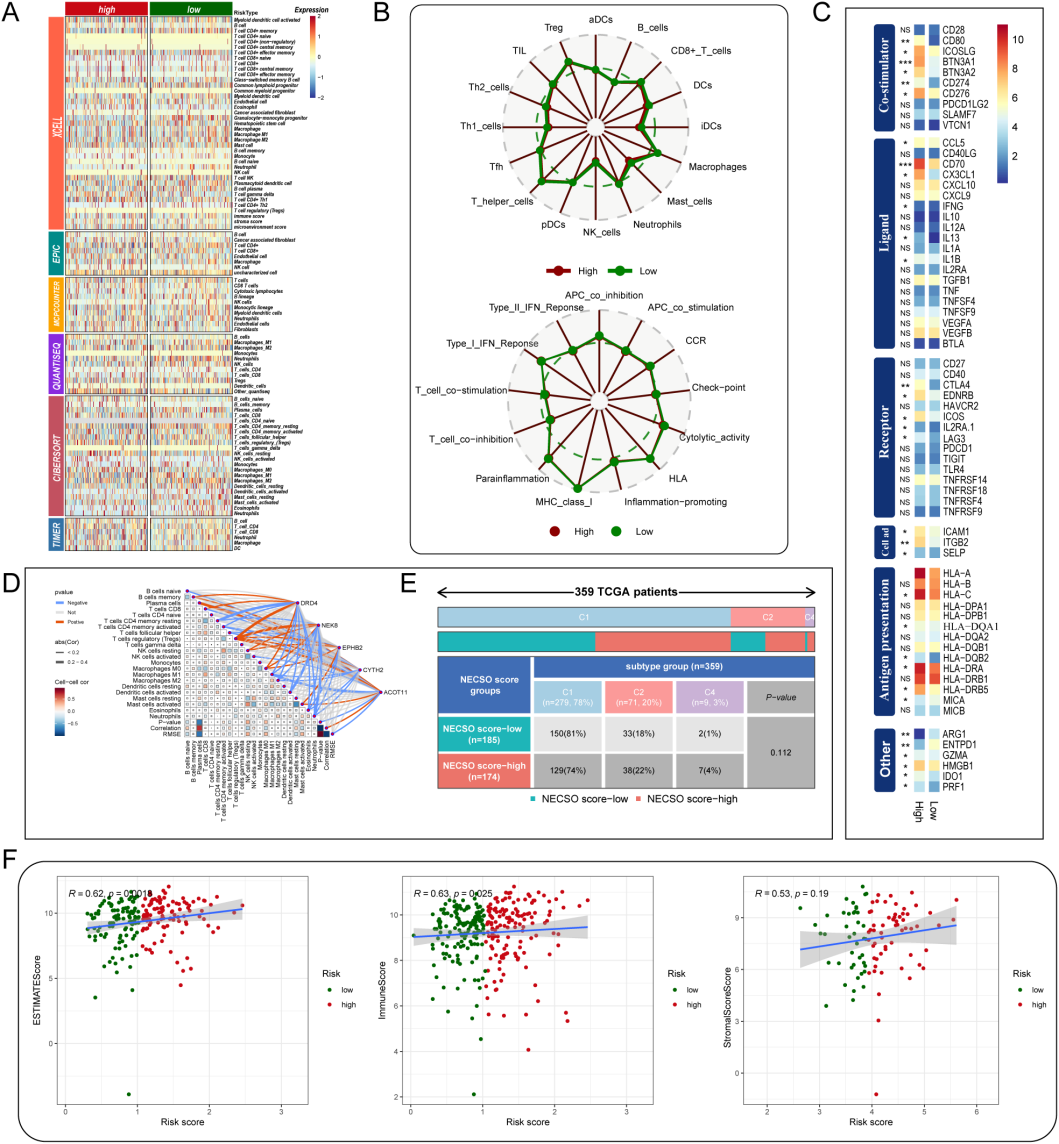
System-level associations between the NECSO prognostic signature and the tumor immune microenvironment. **(A)** Multi-algorithm immune deconvolution heatmap comparing high- and low-NECSO–risk groups using xCell, EPIC, MCP-counter, QUANTISEQ, CIBERSORT, and TIMER (group differences by Wilcoxon rank-sum test). **(B)** Radar plots. Top: differential infiltration of key immune populations (e.g., CD8^+^ T cells, dendritic cells, Tregs). Bottom: pathway/function activity contrasts (APC co-inhibition/co-stimulation, cytotoxic activity, type I/II IFN responses, chemokine signaling, HLA, etc.) between risk groups. **(C)** Immunomodulator atlas heatmap summarizing expression and significance of costimulators, ligands, receptors, adhesion/chemotaxis molecules, antigen presentation (HLA), and other categories across risk strata. **(D)** Correlation matrix/network depicting associations between NECSO signature genes and major immune-infiltration metrics (color encodes direction/strength; *P* values indicated). **(E)** Immune-subtype distribution in the TCGA cohort (n = 359): composition of NECSO low- and high-risk groups across canonical subtypes (e.g., C1, Wound Healing; C2, IFN-γ Dominant) with *P* values for comparisons. **(F)** Scatterplots relating the NECSO risk score to microenvironmental indices: positive correlations with ESTIMATE score (left) and Immune score (middle), and a weaker association with Stromal score (right). Points are colored by risk group; lines show linear fits with Pearson’s *R* and *P*.

To assess differences in the tumor immune microenvironment associated with the NECSO-based risk score, we compared immune-cell infiltration and pathway activity between high- and low-risk groups. In the high-risk cohort, we observed reduced infiltration of key effector populations, including CD8^+^ T cells, dendritic cells, and regulatory T cells, which suggests an immunosuppressive microenvironment. This was further supported by the lower Immune/ESTIMATE scores in the high-risk group, which indicate a reduced presence of immune cells. While Immune/ESTIMATE scores were higher in the low-risk group, indicating a greater overall immune presence, this was associated with a more robust immune response, with higher infiltration of effector immune cells (**Figure 4A**). Pathway analyses further revealed greater activation of antigen-presenting cell (APC) co-stimulation and cytotoxic programs in the low-risk group, with attenuated responses in the high-risk group (**Figure 4B**). These results are consistent with the idea that low-risk tumors maintain a more active immune response, whereas high-risk tumors exhibit immune evasion. In line with this, immunomodulator profiling demonstrated higher expression of costimulatory molecules (e.g., CD28, CD80) and chemokines (e.g., CCL5, CXCL10) in the low-risk group, supporting the notion of a more favorable immune environment (**Figure 4C**).Correlative analyses between NECSO signature genes and immune infiltration highlighted DRD4 as strongly positively associated with T cells and dendritic cells, suggesting a role in shaping antitumor immunity (**Figure 4D**).Immune-subtype stratification further supported these findings, with low-risk tumors predominantly classified as C1 (Wound Healing), a subtype linked to immune activation and tissue repair, while high-risk tumors were enriched for the C2 (IFN-γ Dominant) subtype, which is associated with immune suppression and aggressive disease behavior (**Figure 4E**).Finally, the risk score correlated positively with the ESTIMATE score (R = 0.62, p = 0.018) and Immune score (R = 0.63, p = 0.025), indicating greater immune suppression in high-risk tumors. The correlation with the Stromal score (R = 0.53, p = 0.19) was weaker, further supporting the idea that immune evasion mechanisms play a key role in the poor prognosis of high-risk tumors (**Figure 4F**).

### 3.4 Alteration landscape of NECSO model genes in CRC

**Figure 5:**
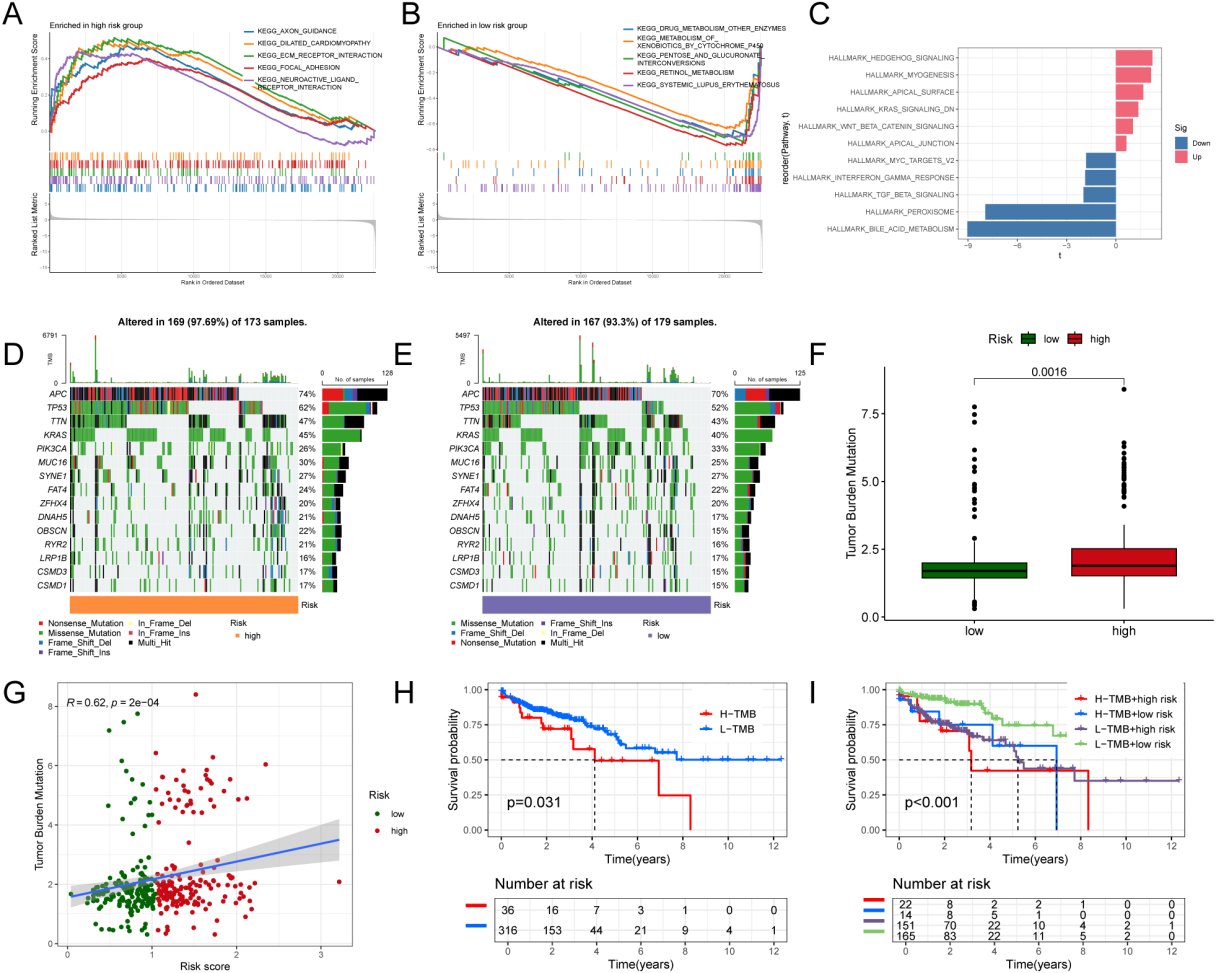
Pathway characteristics, mutational burden, and prognostic relevance of NECSO risk stratification. **(A)** GSEA for the high-risk group showing enrichment of invasion/adaptation pathways (e.g., axon guidance, ECM–receptor interaction, focal adhesion). **(B)** GSEA for the low-risk group, highlighting enrichment of metabolism-related programs (e.g., drug metabolism—other enzymes; pentose and glucuronate interconversions). **(C)** GSVA/Hallmark contrasts: higher activity of Hedgehog, myogenesis, and Wnt/β-catenin signaling in high risk; greater peroxisome and bile acid metabolism in low risk (bar color denotes up/down regulation). **(D, E)** Oncoplots for the top 15 recurrently mutated genes in **(D)** high- and **(E)** low-risk tumors (e.g., APC, TP53, KRAS), with mutation classes indicated. **(F)** Boxplot showing higher tumor mutational burden (TMB) in the high-risk group (*p* = 0.0016). **(G)** Scatterplot demonstrating a positive correlation between risk score and TMB (Pearson *R* = 0.62; *p* = 2×10^-4^); shading indicates 95% CI of the linear fit. **(H)** Kaplan–Meier curves showing worse overall survival for patients with high TMB (*p* = 0.031). **(I)** Combined stratification (TMB × risk), indicating the poorest outcomes in the high-TMB/high-risk subgroup, with risk tables and *p* values shown.

Using GSEA and GSVA, we profiled immune and biological pathway differences between high- and low-risk groups. In GSEA, the high-risk group was significantly enriched for tumor-aggressiveness pathways—axon guidance, ECM–receptor interaction, and focal adhesion—suggesting enhanced invasiveness and adaptive responses. By contrast, the low-risk group showed enrichment of metabolism-related programs (drug metabolism—other enzymes; pentose and glucuronate interconversions), indicative of stronger metabolic capacity and a more active immune milieu (**Figures 5A, B**). Consistently, GSVA revealed preferential enrichment of Hedgehog signaling, myogenesis, and Wnt/β-catenin signaling in the high-risk cohort, whereas the low-risk cohort exhibited higher activity in peroxisome and bile acid metabolism pathways, pointing to heightened metabolic and antioxidant responses (**Figure 5C**). Together, these results suggest that the low-risk group harbors more robust immune and metabolic activity, whereas the high-risk group aligns with invasive phenotypes and immune evasion. Tumor mutational burden (TMB) analyses supported this divergence. In the high-risk group, APC, TP53, and KRAS showed higher mutation frequencies with predominance of missense and frameshift variants (**Figure 5D**), whereas the low-risk group had fewer mutations, largely missense (**Figure 5E**). Boxplots confirmed significantly lower TMB in the low-risk group (p = 0.0016; **Figure 5F**), concordant with a positive correlation between risk score and TMB (R = 0.62, p = 2 × 10^-4^; **Figure 5G**). Survival analyses indicated shorter overall survival for high-TMB patients (p = 0.031; **Figure 5H**), with the worst outcomes in the high-TMB/high-risk subgroup (**Figure 5I**), underscoring the complementary prognostic value of integrating TMB with the NECSO-based risk score.

### 3.5 Enrichment scoring of nuclear NECSO-related genes in scRNA-seq

**Figure 6:**
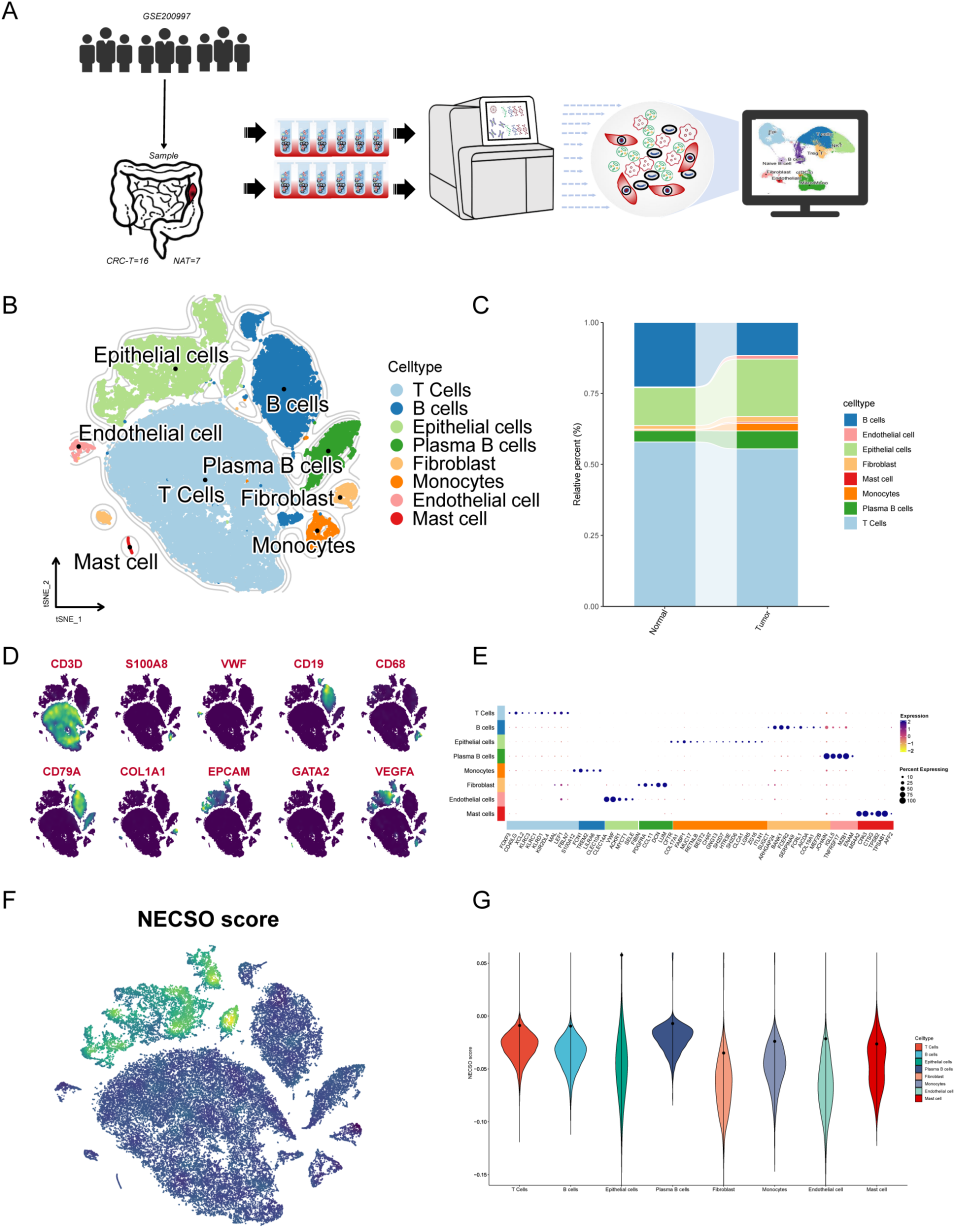
Single-cell characterization of NECSO activity: cellular origins and distribution in colorectal cancer. **(A)** Workflow schematic for the GSE200997 cohort (16 CRC and 7 adjacent normal samples), from dissociation and single-cell RNA sequencing to downstream analysis and visualization. **(B)** t-SNE embedding with cell-type annotation identifying eight major lineages: T cells, B cells, epithelial cells, plasma cells, fibroblasts, monocytes, endothelial cells, and mast cells. **(C)** Stacked bar chart showing relative cell-type composition in normal versus tumor samples. **(D)** Feature maps of canonical markers (e.g., CD3D, S100A8, VWF, CD19, CD68, CD79A, COL1A1, EPCAM, GATA2, VEGFA) overlaid on the embedding to support annotation. **(E)** Dot plot of representative markers across cell types (dot size = percent expressing; color = average expression). **(F)** NECSO module-score feature map computed from the five-gene set (DRD4, NEK8, EPHB2, CYTH2, ACOT11), showing the spatial distribution of NECSO activity at single-cell resolution. **(G)** Violin plots comparing NECSO scores across cell types, indicating predominant enrichment in epithelial cells.

Analyzing the GSE200997 single-cell RNA-sequencing (scRNA-seq) dataset, we profiled 49,589 cells from 16 colorectal cancer (CRC) specimens and 7 adjacent normal tissues (**Figure 6A**). A t-SNE embedding (**Figure 6B**) delineated the cellular landscape, revealing discrete clusters aligned with major lineages. Using canonical markers curated in CellMarker 2.0 (50), we annotated eight principal types: T cells, B cells, epithelial cells, plasma cells, fibroblasts, monocytes, endothelial cells, and mast cells (**Figure 6E**). Comparison of tumor versus normal composition (**Figure 6C**) showed pronounced shifts in immune and stromal compartments: proportions of B, T, and plasma cells changed, with notable increases in monocytes and fibroblasts in tumors, consistent with immune remodeling and stromal activation. Spatial distributions of hallmark genes (e.g., CD3D, S100A8, VWF, CD19, CD68, CD79A, COL1A1, EPCAM) matched the corresponding clusters on the t-SNE map, supporting robust cell-type annotation (**Figure 6D**). To quantify NECSO activity at single-cell resolution, we scored a NECSO gene set (DRD4, NEK8, EPHB2, CYTH2, ACOT11) using AddModuleScore. NECSO scores were elevated predominantly in epithelial cells (**Figures 6F, G**), implicating malignant epithelia as a principal compartment with heightened NECSO-related transcriptional programs.

### 3.6 Heterogeneity of NECSO-related epithelial subpopulations and the GGH+Epi communication hub

**Figure 7:**
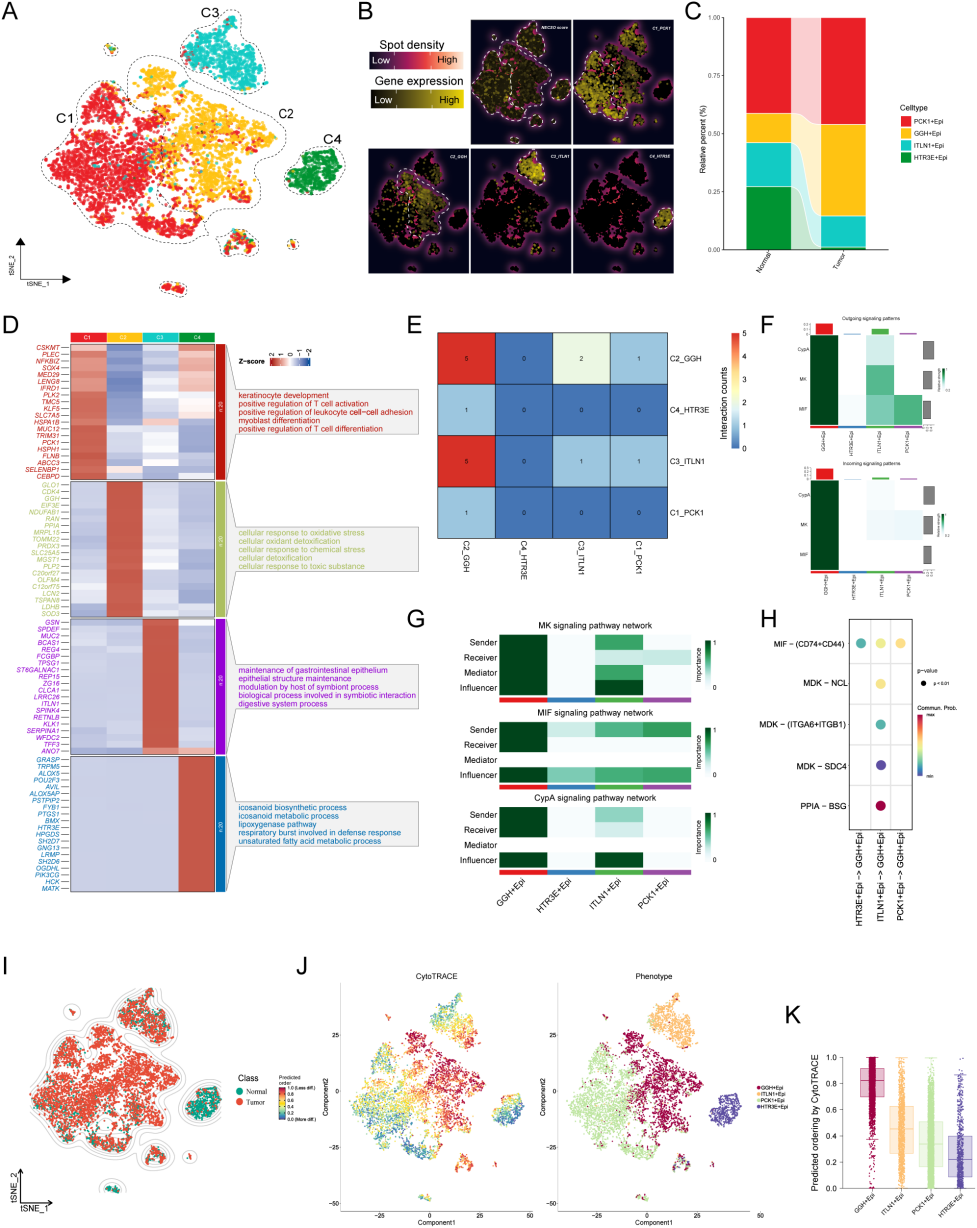
NECSO heterogeneity, communication hub, and differentiation state of epithelial subpopulations. **(A)** t-SNE/UMAP embedding of epithelial cells (n = 7,155) partitioned into four transcriptionally distinct subclusters. **(B)** Subcluster annotation and NECSO module scores: selective marker expression defines subcluster identities; NECSO scores differ markedly across subclusters. **(C)** Composition comparison (tumor vs. normal) showing a pronounced expansion of the PCK1^+^ epithelial subcluster in tumors. **(D)** Functional “fingerprints”: top five GO–Biological Process terms enriched among marker genes per subcluster, indicating divergent functional profiles. **(E)** CellChat-inferred communication strength/number: GGH^+^Epi interacts most frequently with C1_PCK1 and C2_GGH, but less with C3_ITLN1 and C4_HTR3E. **(F)** Signaling pattern analysis highlighting prominent CypA, MK (midkine), and MIF pathway activity in GGH^+^Epi. **(G)** Role decomposition within pathways: GGH^+^Epi acts chiefly as a sender in MK and as a receiver/mediator in MIF/CypA. **(H)** Key ligand–receptor events with high communication probabilities along the GGH^+^Epi axes, including MIF, MDK, and ITGA6+ITGB1 (α6β1 integrin). **(J)** CytoTRACE distribution indicating reduced developmental potential in tumor-derived epithelia relative to normal. **(K)** Boxplots of CytoTRACE scores across subclusters showing the lowest scores in GGH^+^Epi, consistent with a more differentiated/restricted state.

Using scRNA-seq, we isolated 7,155 epithelial cells and partitioned them into four transcriptionally distinct subclusters (**Figure 7A**). Subcluster identities were defined by selective marker expression, and NECSO module scores mapped across subclusters revealed marked inter-subcluster heterogeneity (**Figure 7B**), implying divergent roles of these epithelial states within the tumor microenvironment. Composition analysis (**Figure 7C**) further showed altered epithelial ecology between normal and tumor tissues, with a notable expansion of the PCK1^+^ subcluster in tumors. To characterize function, we identified the top five Gene Ontology biological processes enriched among marker genes per cluster, yielding a molecular “fingerprint” for each (**Figure 7D**). The t-SNE embedding (**Figure 7I**) delineated a clear boundary between tumor-derived epithelia and their normal counterparts. CytoTRACE (**Figure 7J**) indicated reduced developmental potential in tumor epithelia, corroborated by boxplots (**Figure 7K**) showing lower scores—especially in GGH^+^Epi—consistent with a more constrained/differentiated state. We next interrogated epithelial crosstalk with CellChat: GGH^+^Epi displayed frequent interactions with C1_PCK1 and C2_GGH, but fewer with C3_ITLN1 and C4_HTR3E (**Figure 7E**). Signaling-pattern analysis (**Figure 7F**) highlighted prominent CypA, MK (midkine), and MIF activity in GGH^+^Epi. Network decomposition (**Figure 7G**) showed GGH^+^Epi acting chiefly as a sender in MK and as a receiver/mediator in MIF and CypA, underscoring its central role in immune modulation. To improve interpretability, we further prioritized the most significant ligand–receptor interactions that were biologically relevant to immune modulation. Specifically, the top interactions included the MIF axis [e.g., MIF–(CD74+CD44)] and the MDK axis [e.g., MDK–(ITGA6+ITGB1)], both showing high communication probabilities and consistent enrichment in the GGH^+^Epi-centered network (**Figure 7H**). Together, these findings nominate GGH^+^Epi as a pivotal regulator of the tumor immune microenvironment operating through multiple signaling axes and potentially contributing to immune evasion.

### 3.7 Epithelial cell developmental trajectories and NECSO-associated remodeling of cell–cell communication

**Figure 8:**
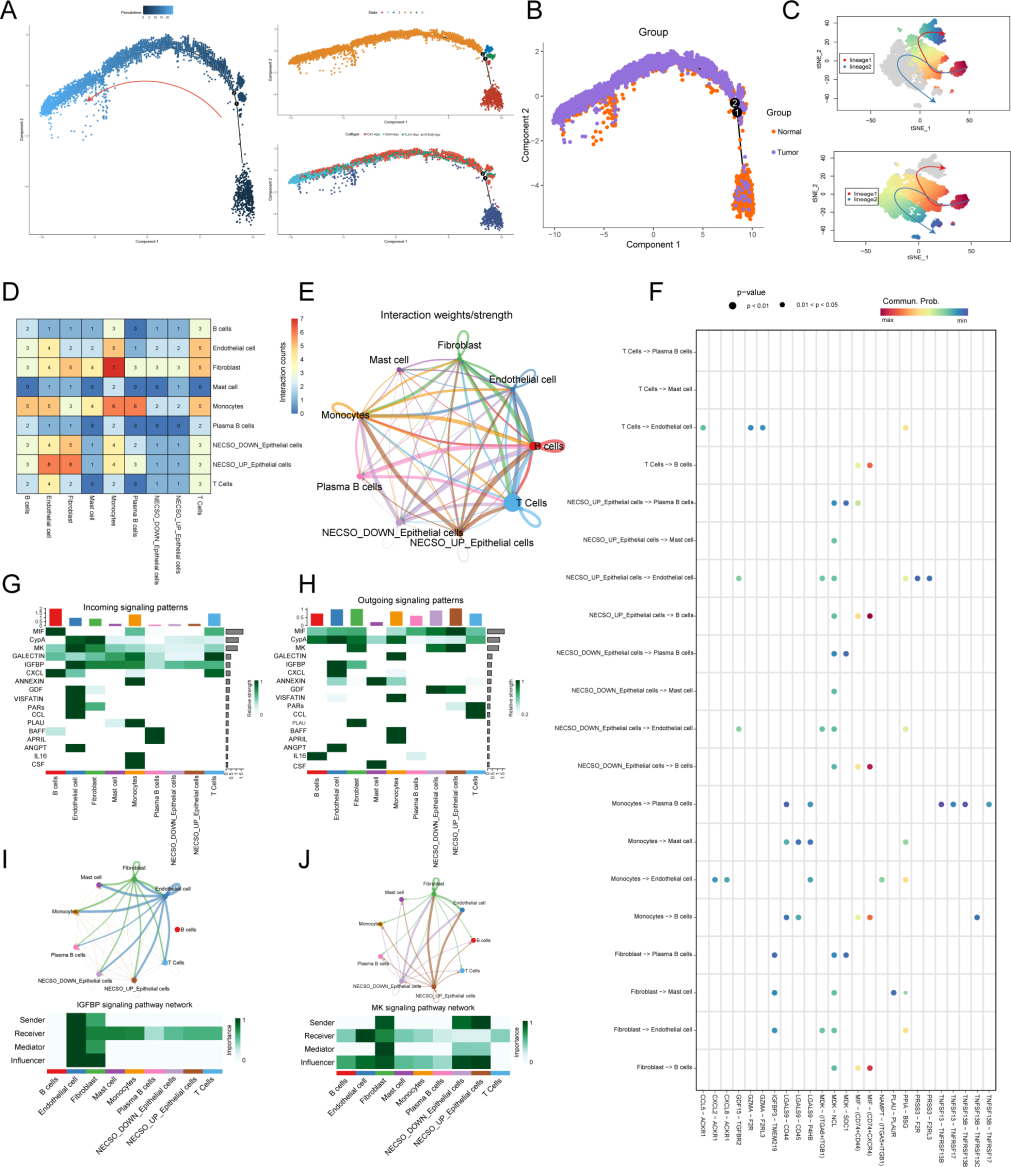
Epithelial developmental trajectories, communication remodeling, and pathway-axis decomposition across NECSO phenotypes. **(A)** Monocle2 pseudotime: epithelial cells progress along a continuous early to late trajectory, terminating in several end-state clusters. **(B)** Pseudotime stratified by tissue of origin: early states are dominated by normal epithelium, whereas late states are enriched for tumor epithelium, indicating downstream malignant states with phenotypic remodeling. **(C)** Slingshot lineage inference: two principal differentiation branches leading to distinct terminal epithelial subtypes; trajectories projected onto UMAP. **(D)** Interaction counts: NECSO_high epithelia engage in significantly more ligand–receptor events with fibroblasts, endothelial cells, and B/T cells than NECSO_low. **(E)** Interaction strengths (weighted sums): NECSO_high exhibits globally stronger communication; NECSO_low shows a preference for monocyte/mast-cell interactions. **(F)** Role decomposition (global): NECSO_high epithelia act primarily as senders, whereas NECSO_low function chiefly as receivers. **(G)** Communication-pattern heatmap: directionality and contribution of key ligand–receptor pathways (arrow width denotes effect magnitude). **(H)** Representative ligand–receptor pairs with high significance differing between NECSO_high and NECSO_low (annotated with *P* values/effect sizes). **(I)** IGFBP axis: NECSO_high epithelia assume the principal sender/influencer role; fibroblasts, endothelial cells, and B cells are receivers/mediators. **(J)** MK axis: concordant directionality with the IGFBP axis, indicating NECSO_high epithelial-driven remodeling of stromal and humoral immune compartments.

Having established the cellular provenance of NECSO signaling and the hub role of GGH^+^Epi, we next charted epithelial dynamics over pseudotime. Monocle2 revealed a continuous trajectory from early to late states, with distinct terminal clusters corresponding to specific epithelial subpopulations (**Figure 8A**). Stratifying by tissue of origin showed early stages dominated by normal epithelia and late stages enriched for tumor cells (**Figure 8B**), indicating that malignant epithelia occupy downstream states with phenotypic remodeling. Slingshot identified two principal differentiation branches leading to divergent terminal phenotypes (**Figure 8C**), consistent with bifurcating trajectories in CRC. To relate NECSO activity to crosstalk within the tumor immune microenvironment (TIME), epithelial cells were stratified by NECSO expression (NECSO_high vs NECSO_low) and ligand–receptor networks reconstructed with CellChat. Overall, NECSO_high epithelia showed more numerous and stronger interactions with fibroblasts, endothelial cells, and B/T cells (**Figures 8D, E**), whereas NECSO_low cells interacted preferentially with monocytes and mast cells. Significant ligand–receptor events mirrored these trends: NECSO_high epithelia acted chiefly as senders, while NECSO_low cells functioned as receivers (**Figures 8F–H**), suggesting that upregulated NECSO programs align with a driver phenotype. Pathway decomposition highlighted the IGFBP and MK (midkine) axes: along both, NECSO_high epithelia assumed the primary sender/influencer role, with fibroblasts, endothelial cells, and B cells as receivers/mediators (**Figures 8I, J**). Collectively, high NECSO expression is associated with a shift from signal reception to emission in epithelial cells, accompanied by directed remodeling of stromal and humoral compartments—implicating NECSO_high epithelia, via the IGFBP and MK axes, in active modulation of the TIME.

### 3.8 Differential expression of NECSO genes and tumor-region–specific spatial enrichment

**Figure 9:**
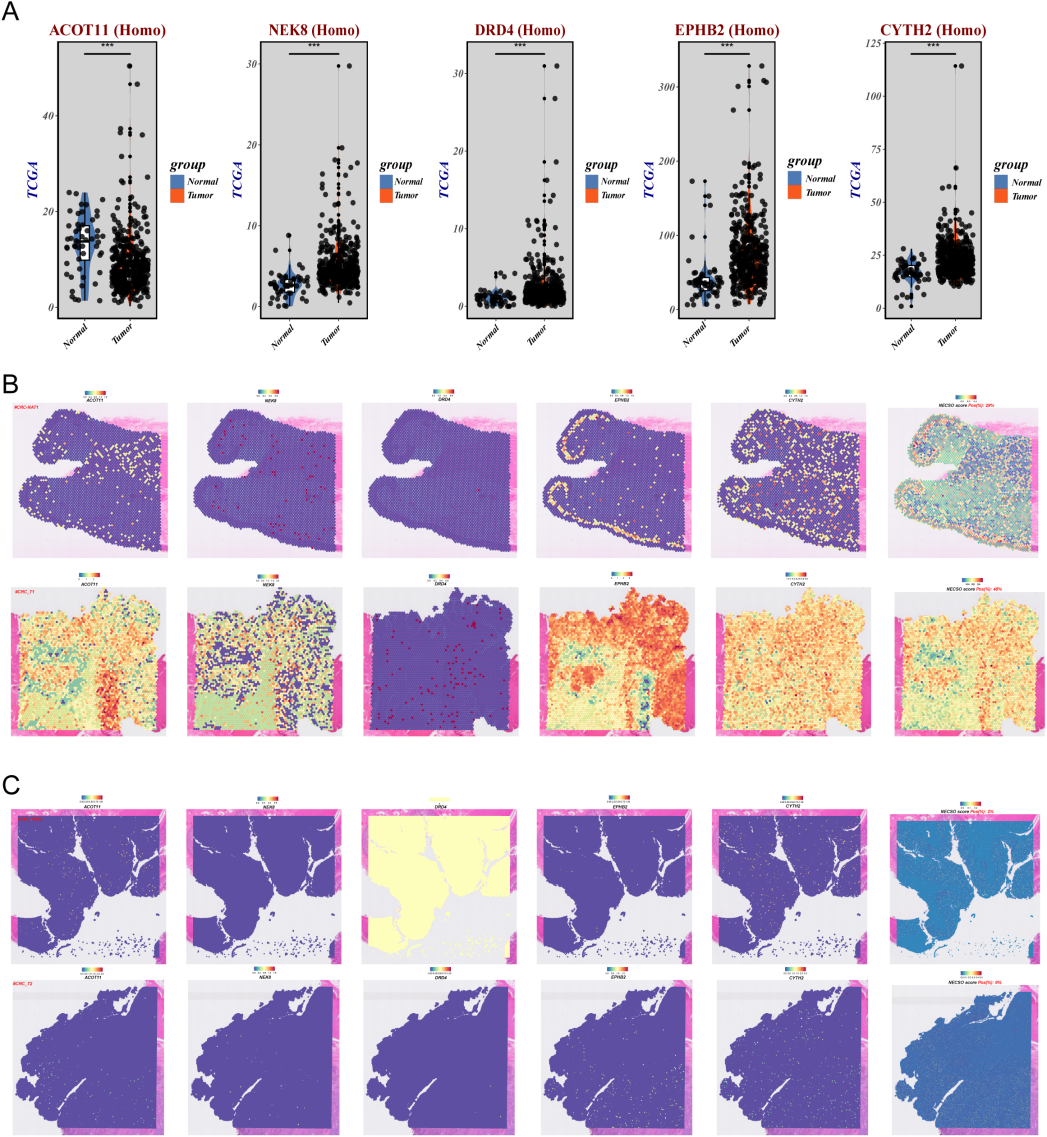
Tumor–normal expression separation and tumor-restricted spatial enrichment of NECSO key genes. **(A)** TCGA cohort: expression of the five genes (NEK8, DRD4, EPHB2, CYTH2, ACOT11). The first four are upregulated in tumors, whereas ACOT11 is downregulated. Box/violin plots show clear separation in dispersion and medians between tumor and normal tissues. **(B)** Spatial transcriptomics, sample 1 (tumor section with matched adjacent normal): NECSO scores are markedly elevated in tumor epithelium with patchy high-score foci at the invasive front; adjacent normal tissue shows uniformly low scores. **(C)** Spatial transcriptomics, sample 2 (higher-resolution tumor section with matched adjacent normal): NECSO scores are markedly elevated in tumor epithelium and increase in extent and intensity with poorer differentiation; adjacent normal tissue shows uniformly low scores.

In the TCGA cohort, analysis of the five NECSO-signature genes (**Figure 9A**) showed NEK8, DRD4, EPHB2, and CYTH2 were broadly upregulated in tumors, whereas ACOT11 was significantly downregulated. Differences in both dispersion and median expression supported a clear tumor–normal separation at the NECSO-related molecular level. To resolve spatial features, we profiled two tumor sections of differing differentiation grades with matched adjacent normals by spatial transcriptomics (**Figures 9B, C**). NECSO scores were markedly elevated within tumor epithelium and increased in extent and magnitude with poorer differentiation; adjacent normals exhibited uniformly lower scores. Concordantly, NEK8/DRD4/EPHB2/CYTH2 showed patchy high expression in tumor epithelium and at the invasive front, colocalizing with NECSO-high regions, whereas ACOT11 was broadly reduced in tumors and comparatively higher in normal glands/epithelium. This pattern reproduced across both specimens, indicating tumor-restricted activation and regionalization of the NECSO program and further supporting the relatively protective profile of ACOT11.

### 3.9 NEK8 upregulation and validation in CRC

**Figure 10:**
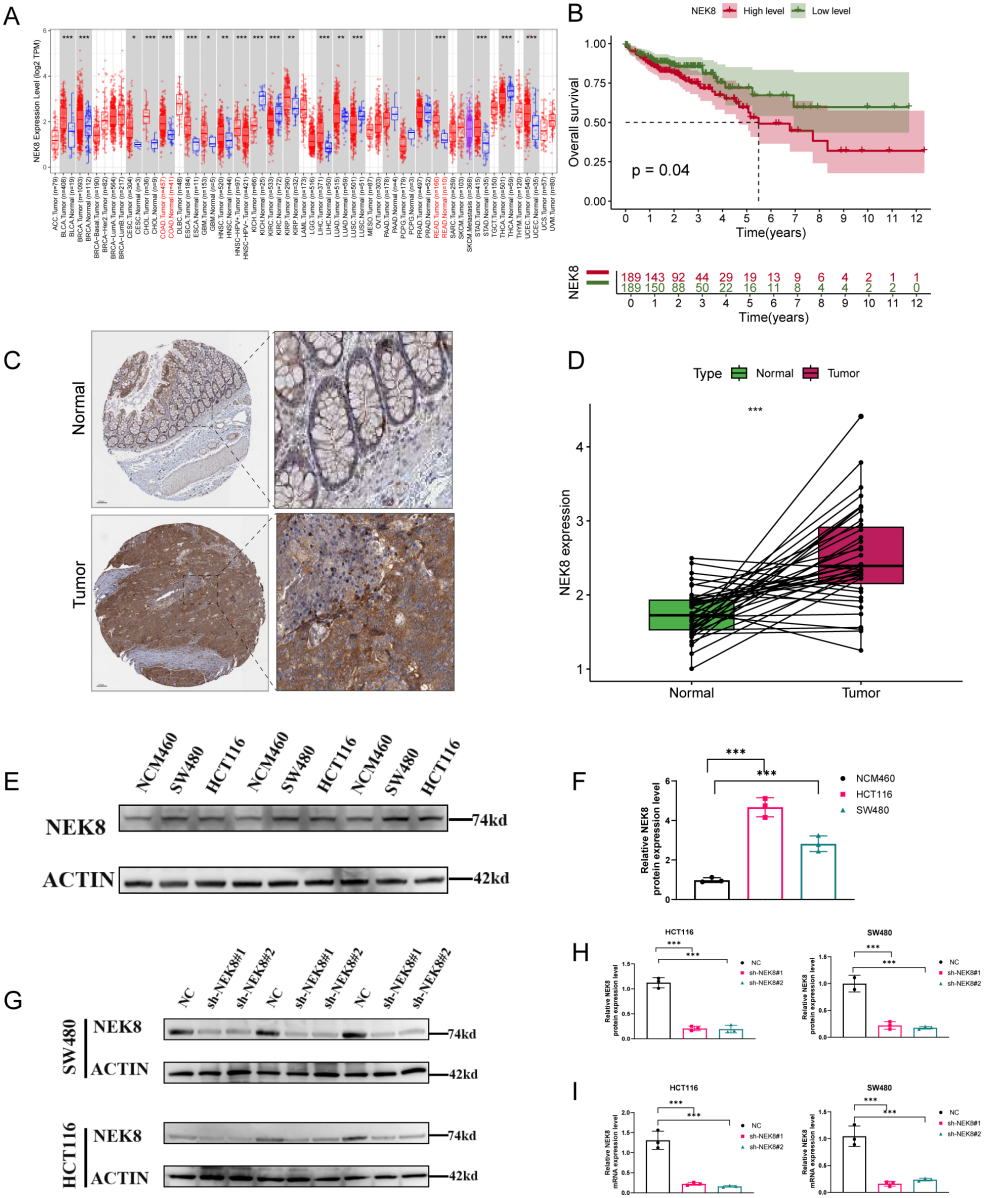
Pan-cancer upregulation of NEK8, prognostic relevance, and expression/knockdown validation in CRC. **(A)** Pan-cancer analysis (TIMER): NEK8 is significantly upregulated in tumors versus adjacent normal tissue across multiple cancer types (P<0.05 in many, some P<0.001), including COAD/READ, STAD, ESCA, LUAD/LUSC, BRCA, HNSC, LIHC, and PAAD. **(B)** TCGA-CRC Kaplan–Meier curves: stratification by median NEK8 expression shows worse overall survival in the NEK8-high group (P = 0.04). **(C)** HPA immunohistochemistry: increased NEK8 protein staining in CRC tissues. **(D)** Paired transcript analysis: tumor samples exhibit significantly higher NEK8 mRNA than matched normal tissue (paired test; P shown). **(E)** Western blot: comparison of NEK8 protein in NCM460 (normal colonic epithelium) versus CRC cell lines (HCT116, SW480). **(F)** Quantification (bar/box): elevated NEK8 protein in CRC cell lines relative to NCM460 (mean ± SEM; P annotated). **(G)** Knockdown validation (WB): two independent shRNAs (sh-NEK8#1/#2) markedly reduce NEK8 protein in HCT116 and SW480 compared with the negative control (NC). **(H)** Densitometry: normalized NEK8 protein levels corresponding to **(G)** (β-actin loading control; P shown). **(I)** qRT–PCR: NEK8 transcripts are significantly decreased in sh-NEK8#1/#2 groups versus NC (2^–ΔΔCt; mean ± SEM; P annotated;β-actin served as the loading control. Significance is indicated as ns, P≥0.05; P<0.05; *P<0.01; **P<0.001; ***P<0.0001.).

To functionally validate the NECSO model genes, we prioritized NEK8 and performed genetic perturbation. A pan-cancer analysis with TIMER showed NEK8 upregulation in most tumors relative to adjacent normal tissue (multiple types p < 0.05; several p < 0.001), including colorectal (COAD/READ), gastric (STAD), esophageal (ESCA), lung adenocarcinoma and squamous carcinoma (LUAD/LUSC), breast (BRCA), head and neck squamous carcinoma (HNSC), liver (LIHC), and pancreatic (PAAD) cancers (**Figure 10A**). In TCGA-CRC, Kaplan–Meier analysis stratified by median NEK8 expression indicated worse overall survival in NEK8-high tumors (p = 0.04; **Figure 10B**). Human Protein Atlas IHC corroborated elevated NEK8 protein in CRC tissues (**Figure 10C**), and paired samples confirmed higher NEK8 mRNA in tumors (**Figure 10D**). *In vitro*, Western blotting showed higher NEK8 protein in HCT116 and SW480 than in normal NCM460 cells (**Figure 10E**), with significant quantification (**Figure 10F**). For functional assays, two independent shRNAs (sh-NEK8#1/#2) were introduced into HCT116 and SW480; both markedly reduced NEK8 protein versus negative control (NC) (**Figure 10G**; densitometry in **Figure 10H**) and achieved concordant transcript knockdown by qRT-PCR (**Figure 10I**).

### 3.10 Functional validation of NEK8

**Figure 11:**
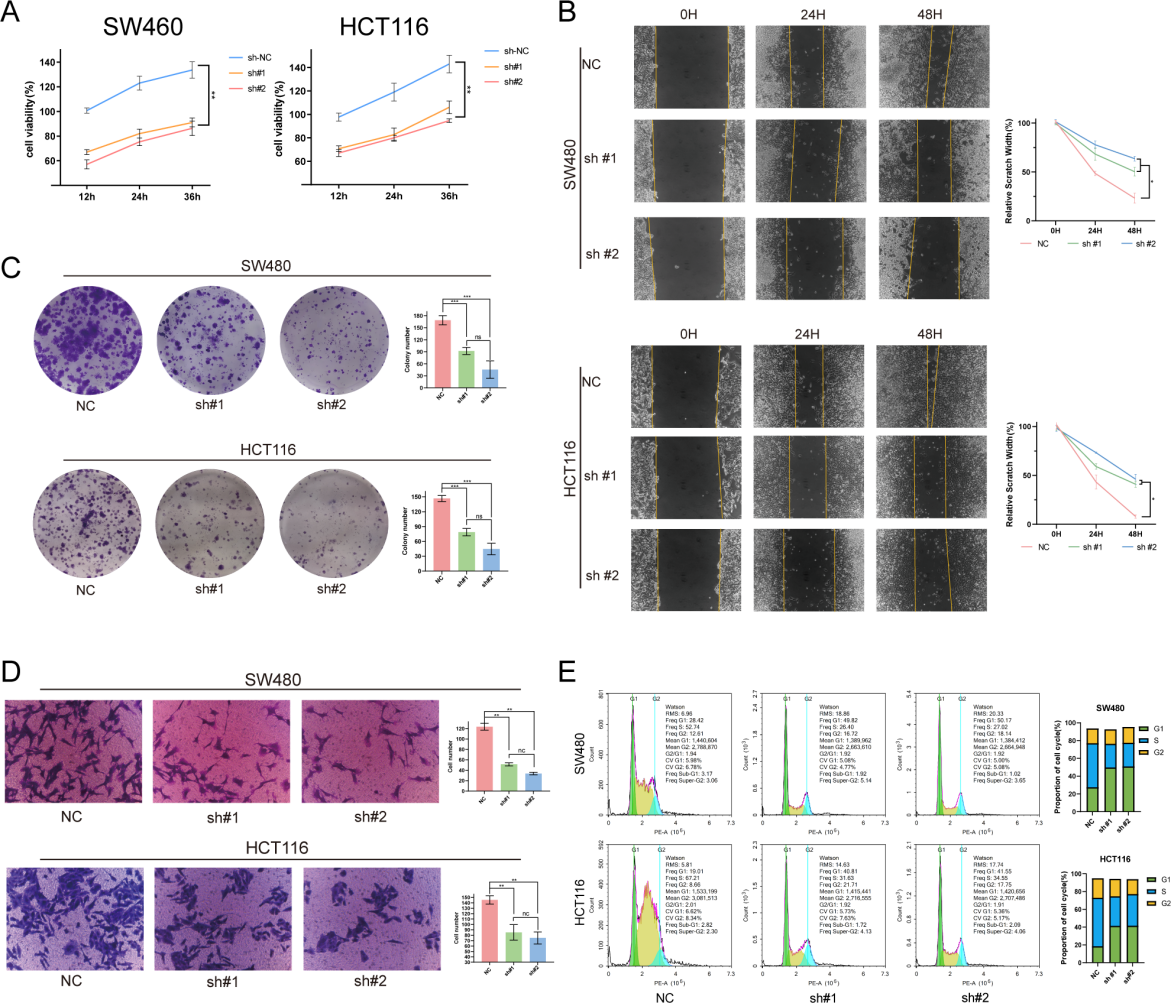
NEK8 knockdown suppresses malignant phenotypes in CRC and induces G1/S cell-cycle arrest. **(A)** Cell proliferation (CCK-8): in HCT116 and SW480 cells, sh-NEK8 (#1/#2) significantly reduces proliferation relative to negative control (NC) at 12–36 h. **(B)** Wound-healing assay: representative images at 0/48 h and quantification show decreased migratory capacity in sh-NEK8 groups. **(C)** Colony formation: after 10 days, sh-NEK8 markedly lowers colony numbers (crystal violet staining and counts). **(D)** Matrigel Transwell: sh-NEK8 significantly reduces the number of migrating/invading cells (representative micrographs and quantification). **(E)** Cell cycle (PI flow cytometry): sh-NEK8 increases the G1 fraction with concomitant decreases in S/G2, indicating G1/S arrest; bar plots depict phase distributions with statistics. (Data are shown as mean ± SEM; significance as annotated ns; *P < 0.05; **P < 0.01; ***P < 0.001).

Having shown that NEK8 is upregulated in CRC and associated with poor prognosis—and after establishing stable knockdown with two independent shRNAs in HCT116 and SW480—we performed phenotypic validation. NEK8 silencing markedly suppressed proliferation (CCK-8, 12–36 h; **Figure 11A**), reduced migratory and invasive capacities (wound-healing and Matrigel Transwell; **Figures 11B, D**), and decreased clonogenicity (**Figure 11C**). Flow cytometry further revealed an increased G1 fraction with concomitant decreases in S/G2, consistent with G1/S arrest (**Figure 11E**). These effects were concordant across both shRNAs and cell lines, indicating that NEK8 drives CRC cell proliferation, migration, and invasion and acts as a key promoter of malignant phenotypes.

### 3.11 Immunotherapy prediction and in-silico validation of drug candidates under NEK8 stratification

**Figure 12:**
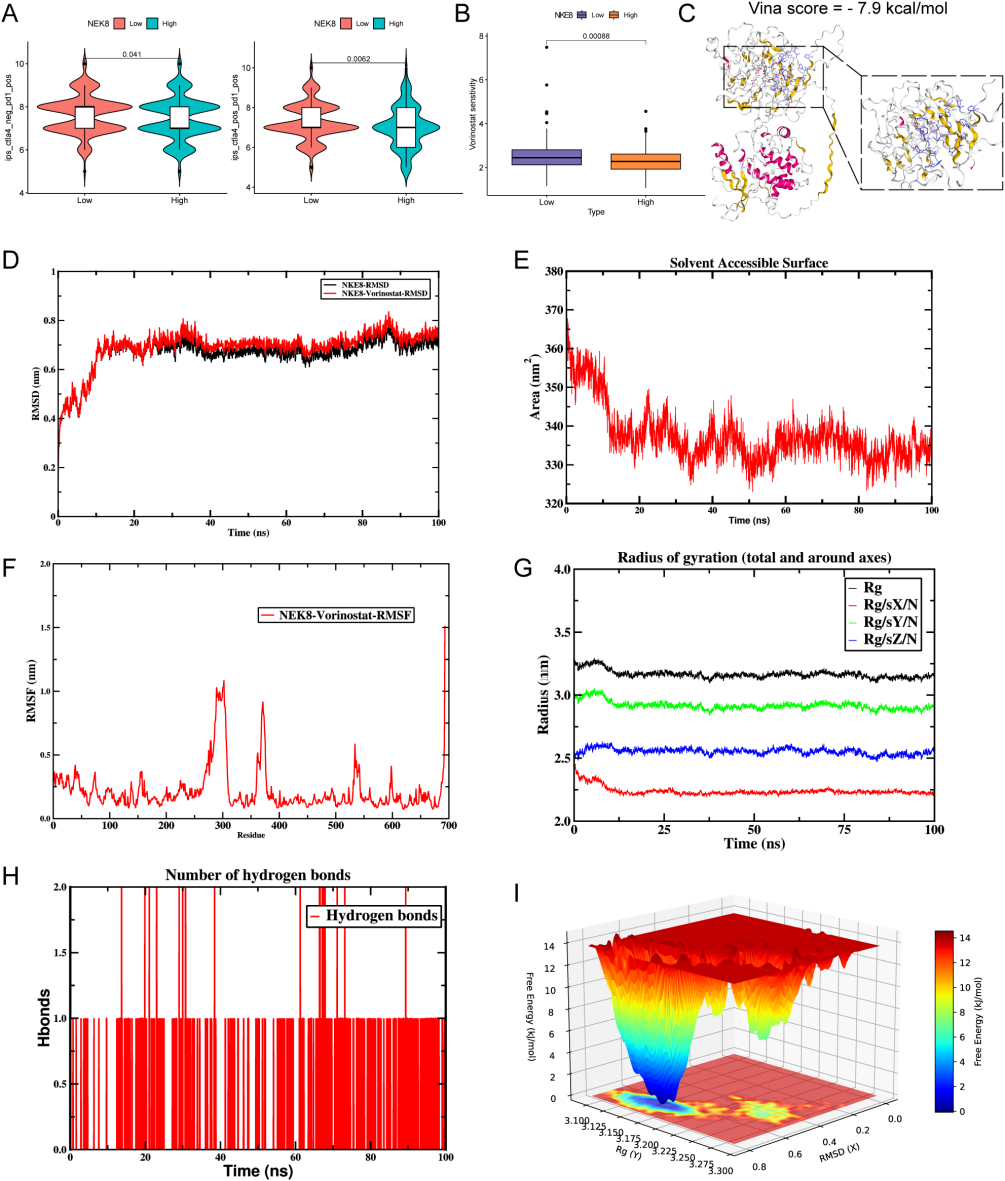
NEK8-based immune stratification, drug sensitivity prediction, and molecular dynamics validation. **(A)** Immunophenoscore (IPS) stratification by NEK8 expression under two immunotherapy contexts. Left: PD-1 monotherapy (CTLA4^-^/PD-1^+^), higher IPS in the NEK8-high group (*p* = 0.041). Right: combined blockade (CTLA4^+^/PD-1^+^), lower IPS in the NEK8-high group (*p* = 0.0062). **(B)** Predicted drug sensitivity (oncoPredict): NEK8-high tumors show reduced sensitivity to vorinostat (*p* = 0.00088). **(C)** Molecular docking (CB-Dock2): representative NEK8–vorinostat binding pose and key contacts; AutoDock Vina score −7.9 kal/mol. **(D)** RMSD over 100 ns: rapid attainment of a stable plateau (~0.6–0.8 nm). **(E)** Residue-level RMSF: highlights flexible regions and stability changes around the binding site. **(F)** SASA vs. time: initial decrease followed by stabilization, consistent with complex compaction. **G** Radius of gyration (R_g): global compactness remains stable during production. **(H)** Protein–ligand hydrogen bonds: persistence of 1–3 H-bonds throughout the trajectory. **(I)** Free-energy surface (FES): a single deep basin indicates a dominant, energetically favorable bound state.

Stratification by NEK8 expression using the immunophenoscore (IPS) revealed divergent immunotherapy-relevant patterns. Under a PD-1 monotherapy context (CTLA4^-^/PD-1^+^), the NEK8-high group showed a significantly higher IPS (p = 0.041), suggesting greater putative responsiveness to PD-1 blockade; conversely, under combined checkpoint inhibition (CTLA4^+^/PD-1^+^), IPS was significantly lower in NEK8-high tumors (p = 0.0062) (**Figure 12A**). However, we acknowledge that these findings are speculative and should be interpreted as hypothesis-generating unless validated in an immunotherapy-treated CRC cohort. Further studies are necessary to verify the predictive value of NEK8 expression for immunotherapy response, including potential confounders like microsatellite instability (MSI) and other CRC-specific factors. In drug prioritization, we focused on vorinostat (SAHA), an FDA-approved oral histone deacetylase inhibitor for cutaneous T-cell lymphoma (51). Within CRC, NEK8-high tumors exhibited reduced in silico sensitivity to vorinostat (higher AUC/IC_50_; p = 0.00088) (**Figure 12B**). Molecular docking indicated favorable NEK8–vorinostat binding (AutoDock Vina score = −7.9 kcal/mol) (**Figure 12C**). Subsequent 100-ns MD simulations supported rapid attainment and maintenance of complex stability, evidenced by plateaued RMSD (~0.6–0.8 nm), decreased then stabilized SASA, constant radius of gyration, persistence of 1–3 hydrogen bonds, and a single deep basin on the free-energy surface (**Figures 12D–I**). Collectively, these findings nominate NEK8 as a biomarker for immunotherapy stratification. Although NEK8-high samples show lower predicted cytotoxic sensitivity to vorinostat, computational evidence of stable binding supports mechanistic follow-up and structure-guided optimization.

## 4 Discussion

Colorectal cancer (CRC) is the third most common malignancy globally and remains a leading cause of cancer mortality. Although chemotherapy, immunotherapy, and targeted agents benefit selected patients, overall prognosis is suboptimal due to pronounced tumor heterogeneity, immune evasion, and therapeutic resistance (52, 53). These challenges underscore the need for novel molecular biomarkers and robust prognostic models to enable individualized therapy and precise risk stratification in CRC (54). In recent years, multi-omics approaches have been increasingly applied to complex diseases, helping to decipher underlying biological mechanisms. Our study integrated bulk transcriptomics with single-cell and spatial transcriptomics to explore NECSO-related pathways in CRC and their clinical significance (55–58).

This study examined sodium-overload cell death (NECSO)–related genes in CRC, derived a prognostic risk score from these genes, and evaluated its immunologic correlates within the tumor microenvironment. Using TCGA-CRC data, we prioritized TRPM4 as the index gene and, based on its expression and co-variation with related transcripts, constructed a NECSO-based risk model. High-risk patients exhibited attenuated immune infiltration—most notably reduced CD8^+^ T cells, dendritic cells, and regulatory T cells—whereas low-risk patients displayed a more active milieu marked by higher immune-cell density and enhanced pathway activation. Thus, the NECSO score not only predicts prognosis but also captures salient differences in the tumor immune microenvironment across strata. Pathway analyses further showed greater activation of antigen-presenting cell (APC) co-stimulation and cytotoxic programs in the low-risk cohort, consistent with a more competent antitumor response, whereas the high-risk cohort displayed blunted immune activity, plausibly reflecting heightened immune evasion. Collectively, these findings support the utility of the NECSO-based model for prognosis and for refining immunotherapeutic strategies by illuminating immune-escape biology in CRC.

The role of NECSO-related genes in the tumor immune microenvironment was further corroborated. Emerging evidence suggests that sodium overload, by activating NECSO programs, is closely linked to immune evasion and therapeutic resistance in tumor cells (9, 59–61). These findings offer a fresh perspective on immune escape and nominate plausible avenues for immunotherapeutic targeting. Notably, the NECSO risk score correlated positively with the ESTIMATE and Immune scores, indicating that the model faithfully captures immune features of the microenvironment. Accordingly, immunotherapy strategies informed by this model may aim to restore or potentiate antitumor immunity by modulating sodium-overload pathways, thereby improving clinical efficacy.

We focused on NEK8, a key component of the NECSO-derived risk signature, whose role in CRC warrants deeper investigation. Never-in-mitosis A–related kinase 8 (NEK8; also NPHP9/NEK12A) is a serine/threonine kinase involved in cell-cycle control, primary-cilium disassembly, and the DNA-damage response (62–65). Prior studies link NEK8 to disease progression and show it enhances gastric cancer cell proliferation and migration; more recently, NEK8 was reported to modulate CRC progression via MYC phosphorylation (66). In our data, NEK8 was significantly elevated in CRC versus normal tissue and inversely associated with overall survival, indicating that NEK8 contributes to CRC progression and may serve as a prognostic biomarker. Functional assays corroborated this: NEK8 knockdown markedly suppressed CRC cell proliferation, migration, and invasion, underscoring its role in sustaining malignant phenotypes. Mechanistically, NEK8 may influence sodium-handling pathways—facilitating sodium overload and triggering NECSO—which could promote malignant transformation and immune evasion. We also observed associations between NEK8 and the tumor immune microenvironment: high NEK8 expression correlated positively with T-cell and dendritic-cell infiltration, suggesting a role in shaping antitumor immunity via effects on immune-cell trafficking or activation within the TME. Elevated NEK8 may also affect immunotherapy responses, nominating NEK8 as a candidate for therapeutic stratification and intervention.

Despite supporting the utility of a NECSO-based gene signature for prognostication and immune microenvironment profiling in CRC, this study has limitations. First, although we analyzed TCGA and GEO datasets, cohort heterogeneity and sampling bias may limit generalizability; validation in large, multicenter clinical cohorts is warranted. Second, while single-cell RNA-seq and spatial transcriptomics were integrated to interrogate NECSO programs in the TME, constraints in spatial resolution and sample size may preclude full delineation of malignant–immune interactions. Finally, although NEK8 was examined in depth in CRC, its mechanistic roles across other tumor types remain insufficiently defined and merit further investigation. However, it is important to note that these results are still in the preliminary stage, and we have not yet conducted direct assays to measure sodium influx or validate NECSO-like cell death phenotypes. Therefore, further studies, including intracellular sodium flux assays and NECSO-like cell death experiments, will be crucial to validate this hypothesis and fully elucidate the role of NEK8 in sodium overload and cell death.

Overall, the NECSO-based risk signature provides a practical tool for prognostic stratification in CRC and a framework to guide the precise deployment of immunotherapies. Future studies should define the mechanistic role of NEK8 in CRC in greater detail—particularly its dual involvement in sodium overload and immune evasion—to expose therapeutic vulnerabilities and inform rational combinations.

## 5 Conclusion

We developed and validated a NECSO-derived five-gene prognostic signature that tracks an immune-cold phenotype, enrichment of invasion pathways, and elevated tumor mutational burden. Single-cell and spatial transcriptomics mapped NECSO activity to malignant epithelium and implicated the IGFBP and MK axes in epithelial-driven remodeling of stromal and humoral immunity. Convergent functional and computational evidence identifies NEK8 as a central node that drives malignancy and shapes antitumor immunity, nominating it for patient stratification and therapeutic targeting. The signature supports risk assessment and immunotherapy selection and motivates combination strategies that modulate sodium homeostasis.

## Data Availability

The original contributions presented in the study are included in the article/**Supplementary Material**. Further inquiries can be directed to the corresponding author.

## Supplementary material

Supplementary Figure 1 Quality control, batch correction, and clustering–resolution assessment for single-cell data. **(A)** QC violin plot showing distributions of three key metrics by cell identity: number of detected genes (*nFeature_RNA*), UMI counts (*nCount_RNA*), and mitochondrial proportion (*percent.mt*). These metrics inform depth, cell integrity, and filtering thresholds. **(B)** Batch-effect evaluation. Two-dimensional embeddings before correction (PCA colored by *orig.ident*) and after correction (Harmony). Increased overlap across samples post-correction indicates effective mitigation of batch effects. **(C)** Clustering-resolution scan (clustree). Tree visualization across a range of Seurat resolutions depicting cluster splits/merges and stability. Node size approximates cell numbers; edges track cluster inheritance between adjacent resolutions. This guides the selection of a robust target resolution for downstream analyses.
