# Supplementary figures and images for: Multi-omics profiling of sodium-overload (NECSO) programs identifies NEK8 as a central driver of colorectal cancer progression through single-cell and spatial transcriptomics
Source: Front Immunol. 2026 Feb 10;17:1765055. doi: 10.3389/fimmu.2026.1765055 (PMC12929418; doi:10.3389/fimmu.2026.1765055)

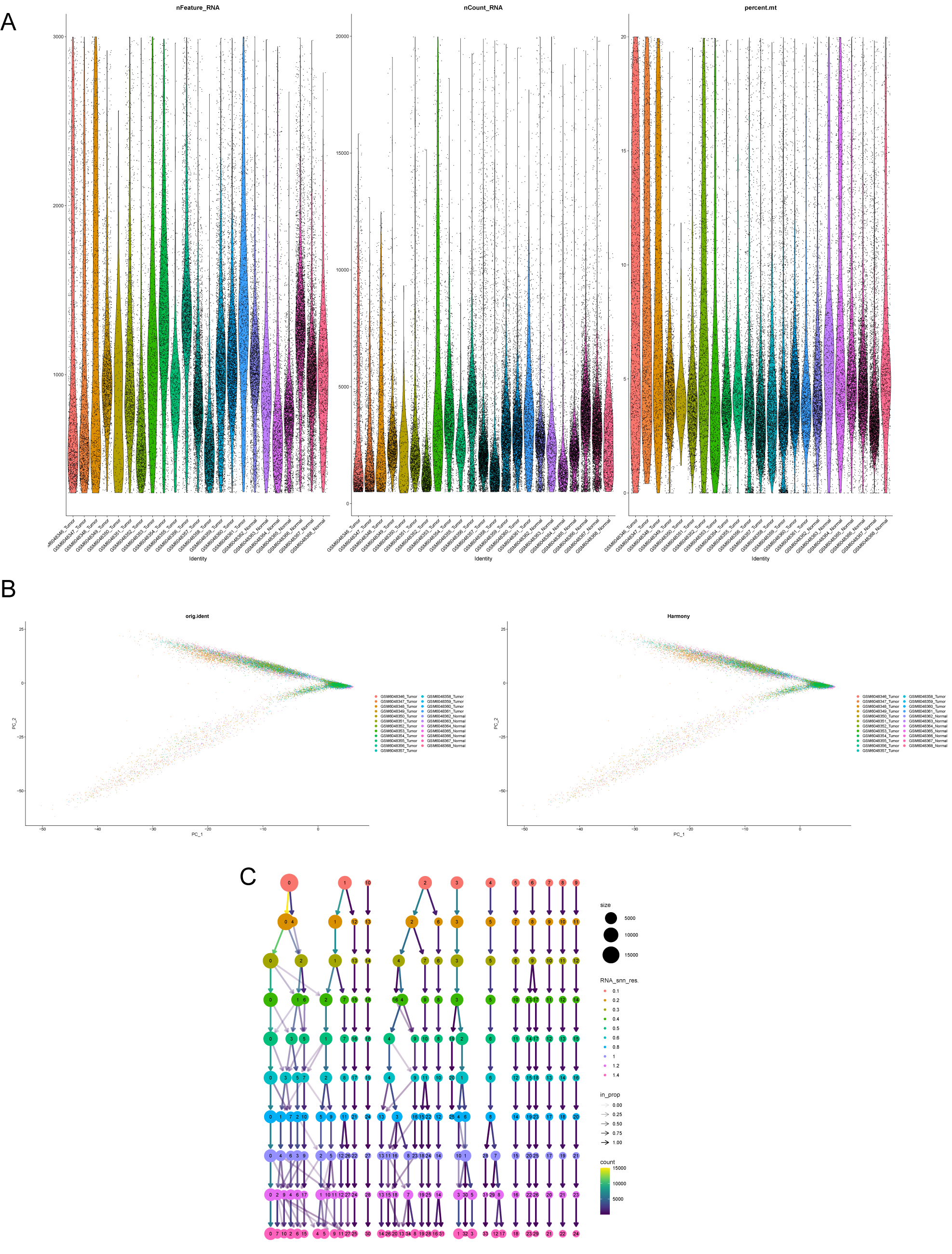

Supplement: Supplementary Figure 1 — Quality control, batch correction, and clustering–resolution assessment for single-cell data. (A) QC violin plot showing distributions of three key metrics by cell identity: number of detected genes (nFeature_RNA), UMI counts (nCount_RNA), and mitochondrial proportion (percent.mt). These metrics inform depth, cell integrity, and filtering thresholds. (B) Batch-effect evaluation. Two-dimensional embeddings before correction (PCA colored by orig.ident) and after correction (Harmony). Increased overlap across samples post-correction indicates effective mitigation of batch effects. (C) Clustering-resolution scan (clustree). Tree visualization across a range of Seurat resolutions depicting cluster splits/merges and stability. Node size approximates cell numbers; edges track cluster inheritance between adjacent resolutions. This guides the selection of a robust target resolution for downstream analyses. [file Image1.tif]
